# The first characterization of multidrug and toxin extrusion (MATE/SLC47) proteins in zebrafish (*Danio rerio*)

**DOI:** 10.1038/srep28937

**Published:** 2016-06-30

**Authors:** Jovica Lončar, Marta Popović, Petra Krznar, Roko Zaja, Tvrtko Smital

**Affiliations:** 1 Laboratory for Molecular Ecotoxicology, Division for Marine and Environmental Research, Ruđer Bošković Institute, Zagreb, Croatia; 2 Oxford Institute for Radiation Oncology, University of Oxford, Oxford, England, United Kingdom; 3 Institute of Molecular Systems Biology, ETH Zurich, Switzerland; 4 Sir William Dunn School of Pathology, University of Oxford, Oxford, England, United Kingdom

## Abstract

Multidrug and toxin extrusion (MATE) proteins are involved in the extrusion of endogenous compounds and xenobiotics across the plasma membrane. They are conserved from bacteria to mammals, with different numbers of genes within groups. Here, we present the first data on identification and functional characterization of Mate proteins in zebrafish (*Danio rerio*). Phylogenetic analysis revealed six Mates in teleost fish, annotated as Mate3–8, which form a distinct cluster separated from the tetrapod MATEs/Mates. Synteny analysis showed that zebrafish *mate* genes are orthologous to human *MATEs*. Gene expression analysis revealed that all the *mate* transcripts were constitutively and differentially expressed during embryonic development, followed by pronounced and tissue-specific expression in adults. Functional analyses were performed using transport activity assays with model substrates after heterologous overexpression of five zebrafish Mates in HEK293T cells. The results showed that zebrafish Mates interact with both physiological and xenobiotic substances but also substantially differ with respect to the interacting compounds and interaction strength in comparison to mammalian MATEs/Mates. Taken together, our data clearly indicate a potentially important role for zebrafish Mate transporters in zebrafish embryos and adults and provide a basis for detailed functional characterizations of single zebrafish Mate transporters.

Absorption, distribution, metabolism and excretion (ADME) are processes that determine the trafficking of compounds through cells and tissues. After entering cells through the cell membrane by uptake transporters or passive diffusion, compounds are further metabolized through the action of phase I and II enzymes and subsequently eliminated from the cell by efflux transporters^1^. The excretion of compounds from the cell to the extracellular fluids is mediated by two transporter families—ATP-binding cassette (ABC) proteins and multidrug and toxin extrusion (MATE) proteins. ABC transporters form a large superfamily of ATP-dependent proteins that mediate the active transport of a vast array of structurally and chemically diverse physiological and xenobiotic substrates across biological membranes. Their role in multidrug (MDR)- and multixenobiotic-resistance (MXR) phenotypes has been extensively studied in mammals and fish^2^. By contrast, despite the fact that the MATE proteins are recognized as another crucial factor in the extrusion of foreign compounds in mammals, knowledge of their roles in non-mammals and/or aquatic organisms is lacking.

MATE/Mate proteins (MATEs in humans, Mates in all other species; gene name *SLC47* in humans, *slc47* in all other species) belong to the superfamily of solute carriers (SLCs)^3^. They function as bidirectional transporters, with the efflux of substrates linked to the proton-coupled electroneutral exchange^4^. MATEs/Mates are 400–600 amino acids long and consist of 12–13 transmembrane domains (TMDs) with an intracellular N terminus and intracellular or extracellular C terminus^5^,^6^. They have recently been characterized in mammals and include three functional proteins in humans (MATE1, MATE2 and MATE2-K)^7^,^8^, three in mice (Mate1a, Mate1b, and Mate2)^9^,^10^,^11^, and two proteins in rats (Mate1 and Mate2)^10^,^12^ and rabbits (Mate1 and Mate2-K)^13^. As Mates have not been investigated thus far in non-mammalian organisms, the classification of this family was only based on mammalian members and includes three groups: group I with human, mouse, rat and rabbit MATE1/Mate1; group II with human and rabbit MATE2/Mate2, also known as MATE2-K; and group III with rat and mouse Mate2^10^,^14^.

Mammalian MATEs/Mates are generally ubiquitously expressed, with the highest expression in the kidney and liver, where they are located in the apical membrane of the proximal and distal renal tubules and in the apical membrane of hepatocytes^15^,^16^. Unlike human MATEs, mouse and rat Mate2 are found only in the testes^10^. Based on their transport function, MATE/Mate proteins are polyspecific transporters that mediate the efflux of cationic compounds, e.g., model cations, such as tetraethylammonium (TEA) and 1-methyl-4-phenylpyridinium (MPP^+^), or weak bases that are positively charged at physiological pH (e.g., cimetidine and metformin)^17^. In some cases, zwitterionic compounds (e.g., cephalexin) and even anionic compounds (e.g., estrone-3-sulfate) can be transported^18^. Recently identified physiological substrates of MATEs/Mates include creatinine, thiamine, guanidine and estrone-3-sulfate, whereas recently identified xenobiotic substrates include antiviral drugs and the antidiabetic drug metformin^3^,^18^,^19^. Known inhibitors of MATEs/Mates include choline, corticosterone, serotonin, and the xenobiotics nicotine and imatinib^3^. Accordingly, the proposed physiological role of MATEs/Mates in mammals is the excretion of drugs, toxins and endogenous metabolites through the kidney and liver^5^,^12^ and possibly testosterone efflux from the Leydig cells in the testes^10^.

Because little is known about non-mammalian Mates, our study was focused on the identification and initial characterization of Mates in zebrafish (*Danio rerio*), a well-established model species in biomedical and environmental research. Our primary goals were (1) to obtain a comprehensive set of phylogenetic and gene expression data to indicate the potential importance of Mates in zebrafish embryos and adults and (2) to determine the interaction profiles of zebrafish Mates with potential substrates as the basis for developing plausible hypotheses related to the physiological and/or defensive roles of these transporters. To position and annotate zebrafish Mates in relation to the other phyla, we performed a detailed phylogenetic analysis of MATEs/Mates from bacteria to mammals. After identification of six zebrafish *mate* genes, synteny analysis was performed in order to elucidate the orthologous relationships with human *MATEs*. Gene expression analysis (qRT-PCR) of the identified zebrafish *mates* was performed in zebrafish embryos at different developmental stages, followed by tissue-specific determinations in adults. Finally, an initial functional characterization of selected proteins was performed using transport activity assays with model substrates after heterologous overexpression of zebrafish Mates in HEK293T cells.

## Methods

### Chemicals

All tested compounds, model fluorescent substrates and interactors alike, were purchased from Sigma-Aldrich (Taufkirchen, Germany) unless stated otherwise.

### Phylogenetic analysis

*Mate* gene sequences were retrieved from The National Center of Biotechnology Information (NCBI) (http://www.ncbi.nlm.nih.gov/), ENSEMBL (http://www.ensembl.org/index.html) and JGI Genome (http://genome.jgi-psf.org). Human, rat and mouse *MATE/mate* sequences were used to perform searches across the genomes of other vertebrate species – chicken (*Gallus gallus*), zebra finch (*Taeniopygia guttata*), anole lizard (*Anolis carolinensis*), Western clawed frog (*Xenopus tropicalis*), West Indian Ocean coelacanth (*Latimeria chalumnae*), zebrafish (*Danio rerio*), stickleback (*Gasterosteus aculeatus*), Atlantic cod (*Gadus morhua*), medaka (*Oryzias latipes*), Japanese pufferfish (*Takifugu rubripes*), jawless fish sea lamprey (*Petromyzon marinus*) and cephalochordate florida lancelet (*Branchiostoma floridae*) – using blastx algorithm^20^. Zebrafish sequences were used to find invertebrate *mates* in tunicates – sea squirt (*Ciona intestinalis*) and marine appendicularian (*Oikopleura dioica*), echinodermate California purple sea urchin (*Strongylocentrotus purpuratus*), hemichordate acorn worm (*Saccoglossus kowalevskii*), mollusks – owl limpet (*Lottia gigantea*) and Akoya pearl oyster (*Pinctada fucata*), annelids – polychaete worm (*Capitella teleta*) and leech (*Hellobdella robusta*), arthropode fruit fly (*Drosophila melanogaster*), nematode roundworm (*Caenorhabditis elegans*), cnidarians – starlet sea anemone (*Nematostella vectensis*) and hydra (*Hydra magnipapillata*), sponge (*Amphimedon queenslandica*), placozoa (*Trichoplax adhaerens*), choanoflagellate (*Monosiga brevicollis*) and yeast (*Saccharomyces cerevisiae*) – using blastx algorithm. Bacterial *mates*, NorM from *Vibrio cholera* and *Escherichia coli* were annotated in the NCBI database.

Members of MATE/Mate family were identified based on two criteria: a blastx hit with threshold value of e < 10^−2^, and the presence of two conserved domains (IPR002528), each 140 amino acid residues long with unknown function, situated at the N terminal part (approx. 50^th^ amino acid position) and middle part of the protein (approx. 260^th^ amino acid position)^21^. BioEdit Software version 7.0^22^ was used for sequence editing, while multiple alignments were performed with Muscle algorithm^23^. Phylogenetic trees were constructed using maximum likelihood method in PhyML version 3.0.1 software^24^, and aLRT test^25^ was used for calculating robustness of the tree using the Seaview4 software^26^.

### Gene expression analysis

Adult zebrafish were bought from a local fish supplier. All experimental procedures involving live fish were carried out in accordance with the directions given in the EU Guide for the Care and Use of Laboratory Animals, Council Directive (86/609/EEC) and the Croatian Federal Act on the Protection of Animals (NN 135/2006). The protocol used in the study was approved by the Bioethics Committee of the Ruđer Bošković Institute, Zagreb, Croatia (Permit Number: BP-1504/2-2011).

Tissue specimens (brain, gills, liver, intestine, gonads and eyes) of five animals were pooled together and stored in RNA later (Qiagen, Hilden, Germany) for subsequent RNA isolation (15 animals were sacrificed for kidney tissue due to the small organ size). All zebrafish specimens that were sacrificed for tissue/RNA isolations were anesthetized by overdose of tricaine methane sulfonate (MS222, 200 mg/L) by prolonged immersion (10 min), followed by one of the established confirmatory methods. Fertilized eggs were collected after spawning, washed and distributed in culture plates where they were developing at 28 °C and 14L:10D cycle. Embryos (3 independent pools, 15–30 embryos per pool per time point from the same spawning group) were collected at time intervals of 1, 4, 6, 12, 24, 48 and 72 hours post fertilization (hpf), in RNA later (Thermo Fisher Scientific, Waltham, USA) and stored at −80 °C until RNA extraction. Tissues were homogenized using rotor-stator homogenizer (IKA, Staufen, Germany) at 10,000 rpm for 20 s. RNA isolation was conducted with Rneasy Plus Kit (Qiagen) which included gDNA eliminator columns, except in the case of eyes, and the embryonic developmental stages when Tri-reagent was used for RNA isolation according to the manufacturer instructions (Sigma-Aldrich, Taufkirchen, Germany) followed by DNase I treatment (Applied Biosystems, Foster City, USA) and final purification with RNeasy Protect Kit (Qiagen). RNA was quantified using BioSpec nano micro-volume spectrophotometer (Shimadzu, Kyoto, Japan) and RNA quality was checked visually by gel electrophoresis. Reverse transcription was carried out with High Capacity cDNA Reverse Transcription Kit with RNase Inhibitor (Applied Biosystems, Foster City, CA, USA). Two additional sets of zebrafish embryonic cDNA (1, 4, 12, 24, 48, 72 and 120 hpf) were kindly provided by Dr. Radmila Kovačević and Branka Glišić from the University of Novi Sad, Republic of Serbia. qRT-PCR primers were designed in Primer Express 3.0 Software (Applied Biosystems) (Supplementary Table S1) and purchased from Invitrogen (Invitrogen, Carlsbad, CA, USA). pGEM-T Vector System I (Promega, Madison, Wi, USA) was used for cloning of 90–110 bp target amplicons (Primer sequences are given in Supplementary Table S2). Isolation and purification of plasmids were performed with QIAprep Spin Miniprep Kit (Qiagen). Sequences were verified by sequencing at the Ruđer Bošković Institute DNA Service (Zagreb, Croatia). Primer efficiencies were determined with qRT-PCR, where recombinant pGEM-T vector carrying target amplicon for respective gene was used as a template. qRT-PCR was conducted as described previously^27^. Relative quantification of genes was carried out with Q-Gene method that includes a correction for differences in amplification efficiencies among genes^28^,^29^ with modification as described previously^30^. Expression thresholds for qRT-PCR data were established as follows: genes were considered to be expressed at low levels if MNE was <499 × 10^7^ (corresponding to C_t_ > 30), at moderate levels when MNE was 500–4,999 (C_t_ = 27, 5–30), at moderately high levels if MNE was 5,000–9,999 (C_t_ = 25, 5–27,5), at high levels if MNE was 10,000–99,999 (C_t_ = 22–25, 5), and at very high levels when MNE was above 100,000 (C_t_ < 22). The described arbitrary thresholds were used to compare relative expression among genes as well as among zebrafish tissues and embryonic developmental stages.

### Cloning and heterologous expression in HEK293T cells

Full-length zebrafish *slc47* genes (Supplementary Tables S2 and S3) were amplified from cDNA from the respective tissue with high fidelity Phusion DNA polymerase (Finnzymes, Vantaa, Finland). The resulting amplicons were cloned into pJET 2.0 vector (Invitrogen, Carlsbad, CA) and the sequences were verified at the Ruđer Bošković Institute DNA Service (Zagreb, Croatia). All genes except *mate5* were subcloned into the expression vectors pcDNA3.1 and pcDNA3.1/His (Invitrogen, Carlsbad, CA). Despite several attempts we were not able to subclone *mate5* in appropriate mammalian expression vector due to its toxicity to *E. coli* DH5α cells.

HEK293T cells were transiently transfected as described previously^31^ using the PEI (polyethyleneimine) reagent in 48-well plates. Cells were in parallel transfected with pcDNA3.1/His/LacZ plasmid and transfection efficiency was evaluated 24 h after transfection with the LacZ staining protocol. Transport experiments were conducted 24 h post transfection, when transfection efficiency was above 70%.

### Transport activity determinations

Six fluorescent compounds were tested as putative Mate substrates: 4′,6-diamidino-2-phenylindole (DAPI), ethidium bromide (EtBr), 4-(4-(dimethylamino)styryl)-N-methylpyridinium iodide (ASP^+^), rhodamine 123 (Rh123), amiloride and berberine. HEK293T cells overexpressing respective single zebrafish Mate transporter were used for further experiments. Cell uptake of all substrates except DAPI was measured in sodium based transport medium (145 mM NaCl, 3 mM KCl, 1 mM CaCl_2_, 0.5 mM MgCl_2_, 5 mM glucose and 10 mM Tris, pH 8.4). Briefly, after 5 minute incubation period, the cells were rapidly washed two times with ice-cold phosphate-buffered saline (PBS), lysed in 250 μL of 0.1% sodium dodecyl sulphate for 30 min and transferred to the black 96-well plates where fluorescence was measured using the microplate reader (Infinite M200, Tecan, Salzburg, Austria) at following excitation and emission wavelengths: ASP^+^, 450/590 nm; EtBr, 535/590 nm; Rh123, 485/530 nm; berberine, 355/540 nm; amiloride, 366/418 nm. For the purpose of DAPI transport assay optimization, screening of interactors, and IC_50_ determination, uptake kinetics in the potassium based transport medium (130 mM KCl, 2 mM K_2_HPO_4_, 1.2 mM MgSO_4_, 1 mM CaCl_2_, 5 mM glucose and 10 mM hepes, pH 7.4) was evaluated within the 5–10 min period depending on the protein, according to the modified protocol established previously by Yasujima *et al*.^32^. DAPI fluorescence was measured at 360 and 460 nm emission and excitation, respectively. The uptake into vector-transfected HEK293T cells (mock cells) was subtracted to obtain transporter-specific uptake.

Calibration curves for all substrates were generated in 0.1% SDS and in the cell matrix dissolved in 0.1% SDS. Resulting linear calibration curves were the same in the SDS and in the dissolved cell matrix. Using the calibration curves, uptake was expressed as pmol of substrate per mg of protein per minute. Total cell protein content was determined according to the Bradford method^33^.

Inhibition assays using DAPI as a fluorescent substrate were performed in the same transport medium. The applied DAPI concentration was 1 μM for Mate4, 6 and 8, and 2.5 μM for Mate3 and 7. Compounds tested for interaction were checked using at least four concentrations but only a representative concentration (with the highest response or the largest concentration in solubility range) is shown. When interacting compounds showed inhibition of DAPI uptake above 30% for at least one Mate protein, IC_50_ values were determined. Compounds with IC_50_ of 1–29 μM were designated as strong interactors, IC_50_ of 30–99 μM indicated moderate interaction, IC_50_ of 100–999 μM was classified as weak interaction and substances with IC_50_ above 1000 μM were considered very weak interactors. Similarly, substrate affinity (*K*_m_) was considered to be very high for *K*_m_ < 1 μM, high for *K*_m_ = 1–29 μM, moderate for *K*_m_ = 30–99 μM, and low for *K*_m_ > 100 μM.

### Western blotting and immunocytochemistry

Cells were collected from 25 cm^2^ culture flasks 24 h after transfection, lysed in RIPA buffer (NaCl 150 mM, EDTA 1 mM, Tris 25 mM, NP-40 0.8%) with protease inhibitors cocktail AEBSF (Sigma-Aldrich, Taufkirchen, Germany) for 30 min on ice, subjected to 3 freeze/thaw cycles, briefly sonicated and centrifuged at 1,000 × *g* for 10 min at 4 °C. Protein concentration was measured using the Bradford method^33^. Part of the Mate6 total cell lysate was subjected to deglycosylation procedure: 50 μg of protein was treated with PNGase F enzyme (Sigma-Aldrich, Taufkirchen, Germany) according to the manufacturer’s protocol.

Proteins (4 μg per lane) were separated by electrophoresis in 10% polyacrylamide gel with 0.1% sodium dodecyl sulphate added. The proteins were then transferred to the polyvinylidene difluoride membrane (Millipore, MA, US) via wet transfer (1 hour at 80 V, without SDS). Blocking was performed in blocking solution containing 5% low fat milk, 50 mM Tris, 150 mM NaCl and 0.05% Tween 20 for one hour. Membranes were washed and incubated for 2 h with anti-His antibody (Clontech Laboratories, CA, USA) diluted 5,000×. Goat anti-mouse IgG-HRP (diluted 15,000×) was used as secondary antibody with one hour incubation period (Santa Cruz Biotechnology, CA, USA). The proteins were visualized by chemiluminescence (Abcam, Cambridge, UK). Protein size was estimated with a protein marker (Bio-Rad Laboratories, CA, USA). For immunofluorescence detection, HEK293T cells were grown on glass coverslips in 24-well culture plates. Twenty four hours after transfection, cells were fixed with 4% paraformaldehyde in PBS during 30 min, washed three times in 100 mM glycin/PBS, permeabilized for 15 min in methanol and blocked in 5% low fat milk for 30 min, with gentle agitation before the first step of the double staining protocol. Coverslips were transferred on microscope slides and incubated with Na, K-ATPase anti-mouse primary antibody (Santa Cruz Biotechnology) (1:150) in blocking solution for 1 h at 37 °C in humidity chamber, washed and then incubated with Cy3-conjugated anti-mouse IgG-HRP as a secondary antibody (1:200) (Santa Cruz Biotechnology) in blocking solution for 1 h at 37 °C. Washing and blocking steps were repeated and coverslips were again transferred on microscope slides and incubated with anti-His antibody (1:100) in blocking solution for 1 h at 37 °C in humidity chamber, washed and then incubated with secondary FITC antibody (1:100) in blocking solution for 1 h at 37 °C. DAPI staining was performed for 45 min at 37 °C in 300 nM DAPI/PBS. After mounting with Fluoromount (Sigma-Aldrich, Taufkirchen, Germany), immunofluorescence was detected using confocal microscope Leica TCS SP2 AOBS (Leica Microsystems, Wetzlar, Germany).

### Data analysis

All assays were performed in 3–5 independent experiments run in triplicates. Data shown on related figures represent mean ± standard errors (SE) or standard deviations (SD). All calculations were performed using GraphPad Prism 5.00 for Windows (GraphPad Software, San Diego, California, USA) as described below. The kinetic parameters, *K*_*m*_ and *V*_*max*_ values were calculated using the Michaelis-Menten equation,


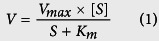


where *V* is velocity (picomoles of substrate per milligram of proteins per minute), *V*_*max*_ is maximal velocity, [*S*] is substrate concentration and *K*_*m*_ is the Michaelis Menten constant. The uptake into vector-transfected HEK293 cells was subtracted to obtain transporter-specific uptake.

For the purpose of IC_50_ calculations, data were fitted to the sigmoidal four-parameter dose-response model (variable slope),


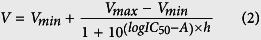


where *V* is response, *V*_*min*_ represents minimum of response, *V*_*max*_ represents maximum of response, *h* is Hill slope parameter, IC_50_ is the concentration of inhibitor that corresponds to 50% of maximal effect, and *A* is the concentration of a tested compound.

## Results

### Phylogenetic and synteny analysis

**Figure 1:**
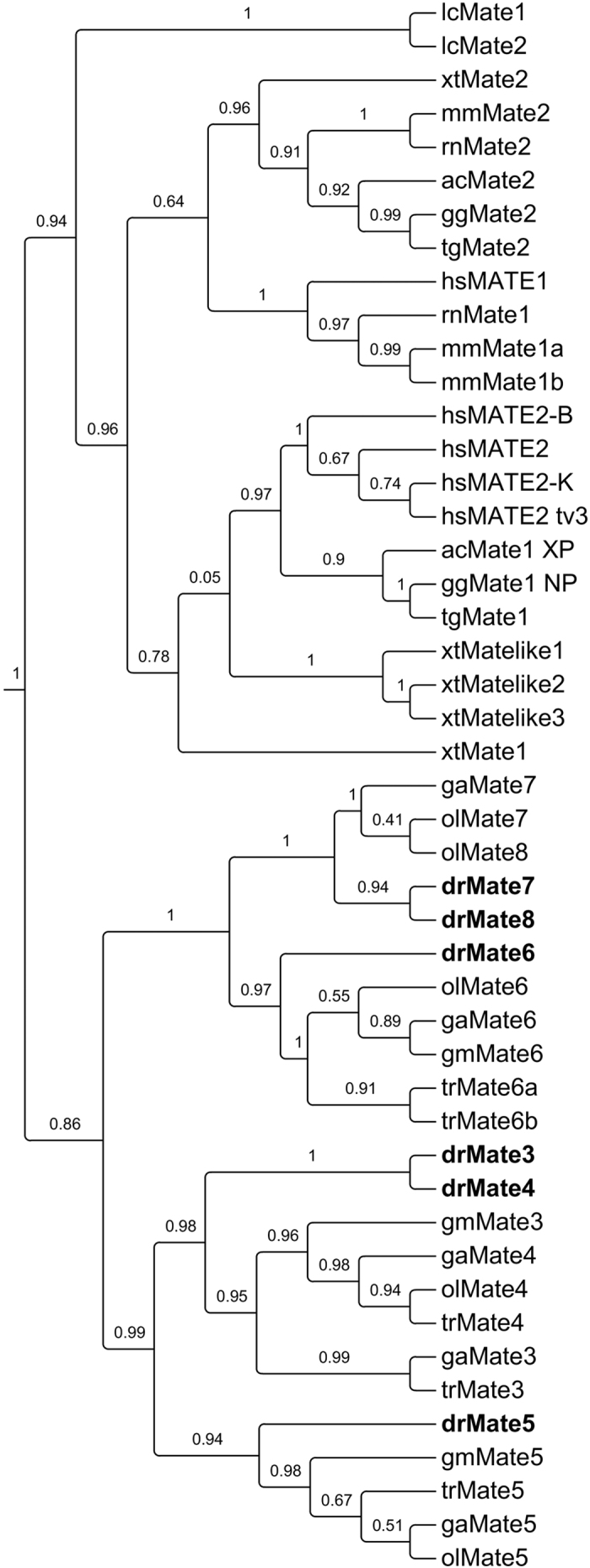
Phylogenetic analysis of MATE/Mate family in vertebrates. Species abbreviations: Hs, *Homo sapiens*; Rn, *Rattus norvegicus*; Mm, *Mus musculus*; Gg, *Gallus gallus*; Tg, *Taeniopygia guttata*; Ac, *Anolis carolinensis*; Xt, *Xenopus tropicalis*; Dr, *Danio rerio*; Tr, *Takifugu rubripes*; Ga, *Gasterosteus aculeatus*; Ol, *Oryzias latipes*; Gm, *Gadus morhua*; Lc, *Latimeria chalumnae*.

Our phylogenetic analysis of the MATE/Mate family included vertebrate and invertebrate phyla. In sum, a total of 63 sequences were identified, of which 54 were annotated as novel MATE/Mate proteins. The resulting phylogenetic tree is presented in Fig. 1, and the provisional annotations and accession numbers are given in Supplemental Table S3. The phylogenetic tree showed the separation of the vertebrate MATE/Mate cluster and invertebrate and protozoan Mates (Supplemental Fig. S1). Within the vertebrate cluster, two subclusters can be distinguished: tetrapod MATEs/Mates and teleostean Mates (Fig. 1). The Mate1 and Mate2 orthologs in a sarcopterygian, the coelacanth *Latimeria chalumnae*, cluster together with amphibian and higher vertebrate MATEs rather than with teleostean Mates. The cephalochordate Mates, represented by the Florida lancelet (*Branchiostoma floridae*), cluster at the root of vertebrate MATE/Mate clusters, whereas invertebrate Mate proteins form a distinct group separated from vertebrate and prokaryotic proteins (Supplemental Fig. S1). Mate sequences were not found in sea lamprey (*P. marinus*), sea squirt (*C. intestinalis*), marine appendicularian (*O. dioica)*, sea urchin (*S. purpuratus*), Akoya pearl oyster (*P. fucata*), leech (*H. robusta)*, fruit fly (*D. melanogaster*), roundworm (*C. elegans*) and hydra (*H. magnipapillata).* Mates in yeast and bacteria form a separate cluster (Supplemental Fig. S1). Unlike tetrapods, which have 1–3 MATE/Mate proteins, teleosts have 3–6 Mates. Accordingly, we identified six Mate proteins in zebrafish and annotated them as Mate3–8 (Fig. 1). Mate3 and Mate4 are the most similar in terms of protein sequences and share 81% amino acid identity (and 43–59% amino acid identity with other zebrafish Mates). Zebrafish Mates share 40–52% protein identity in comparison to human and other mammalian MATEs/Mates, 40–59% to birds (*T. guttata* and *G. gallus*), 40–50% to the anole lizard, 35–51% to the amphibian *X. tropicalis*, 41–70% to the other teleostean Mate proteins and 40–58% to Mate members in a primitive tetrapod, the West Indian Ocean coelacanth (*L. chalumnae*). Zebrafish Mates share 34–46% protein identity with a primitive fish, the Florida lancelet (*B. floridae*), 29–37% with the primitive chordate acorn worm, 29–40% amino acid identity with other invertebrates, 28–36% with protozoans (*T. adherens* and *M. brevicollis*), 24–27% with yeast, and 20–26% amino acid identity with bacterial Mates.

**Figure 2:**
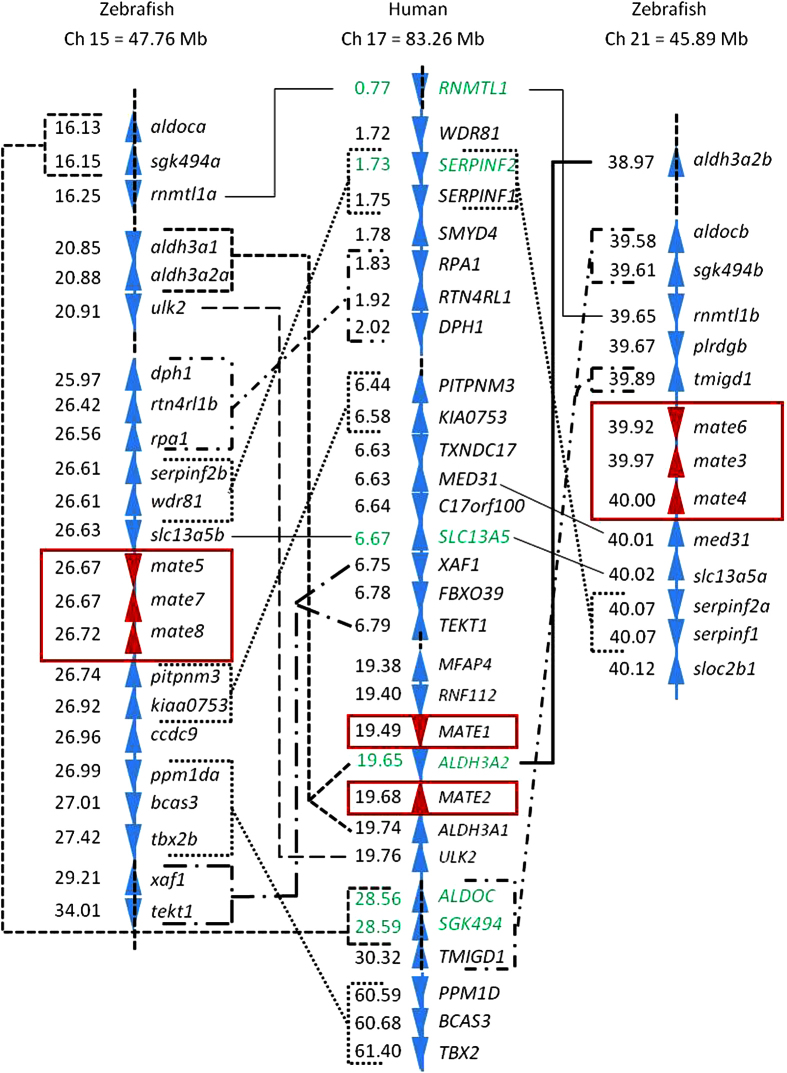
Conserved synteny analysis of zebrafish and human *SLC47/Slc47* genes. Numbers next to the gene names represent megabase pair (Mbp) of gene location on the chromosome. Chromosome segments are represented with blue (continuous segments) and black dashed (discontinuous segments) lines. Orthologs are connected with lines of different style. Human genes with orthologs on both zebrafish chromosomes are shown in green.

Zebrafish Mates are subdivided into two subgroups named Mate3–5 and Mate6–8. One-to-one orthologous relationships to mammalian MATEs/Mates could not be established because fish Mates are more similar within the group than to the mammalian MATEs/Mates clusters. However, our synteny analysis revealed that fish Mate proteins are orthologs of human MATE1 and MATE2. Zebrafish *mate3, 4* and *6* are located on chromosome 21 (ch21), and *mate5, 7* and *8* are located on chromosome 15 (ch15) (Fig. 2). The immediate gene environment of *mate3, 4* and *6* contains upstream (*aldocb, sgk494b, tmigd1*) and downstream (*med31, slc13a5a, serpinf2a*, etc.) genes that are orthologous to the human counterparts in the vicinity of MATE1 and 2 on human chromosome 17. Likewise, *mate5, 7* and *8* have upstream (*aldoc1, sgk494a, dph1*, etc.) and downstream genes (*pitpnm3, kiaa0753, ppm1da*, etc.) that are also orthologous to human ch17. Human genes marked in green in Fig. 2 (*RNMTL1, SERPINF2, SLC13A5, ALDH3A2, ALDOC, SGK494*) have co-orthologous genes on zebrafish ch15 and ch21, revealing double conserved synteny (DCS) blocks that further suggest that zebrafish *mates* are syntenic to the human *MATE* genes (Fig. 2).

### Gene expression analysis

**Figure 3:**
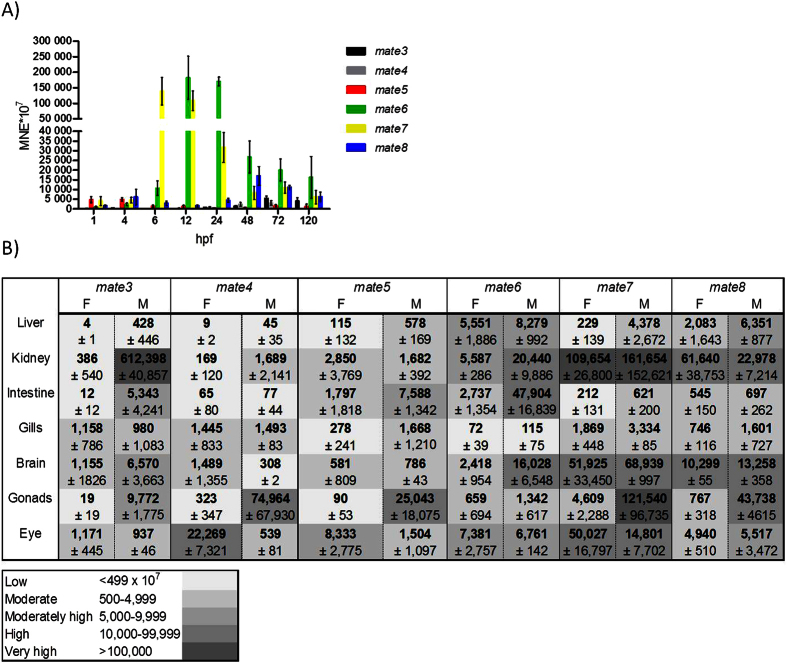
Expression pattern of *Slc47* genes in embryos and tissues of adult zebrafish. (**A**) *Mate* genes in early developmental stages of zebrafish embryos. Results of three to five independent determinations for each developmental stage (three to five pools of 15–30 embryos) are presented, except for 120 hours post fertilization (hpf) where two independent experiments were performed. Data represent MNE (mean normalized expression) ± SE normalized to the *Ef1α*. (**B**) *Mate* genes in specific tissues of female and male zebrafish, quantified with qRT-PCR. Results of two to three independent experiments for females and males (two to three pools of five individuals) are given, while the kidney expression data results from two pools of 15 individuals. Data represent MNE ± SE normalized to the *Ef1α*.

The embryonic and tissue-specific gene expression data for zebrafish *mate* genes are shown in Fig. 3A,B, and additional graphical presentation of the tissue expression data are provided in the Supplementary material (Fig. S2). The expression levels of zebrafish Mates are described in the following paragraphs and were compared based on the expression thresholds for qRT-PCR data described in the Methods section. The expression of *mate* genes in zebrafish embryos in the early developmental stages showed differential expression of specific genes (Fig. 3A). At the first two time points (1 and 4 hpf, respectively), all genes showed low to moderate expression, probably indicating maternal transfer. With the onset of embryonic transcriptional activity at 6 hpf, a significant activation of two genes (*mate6* and *mate7*) was observed, suggesting important roles in embryonic development. Between the finish of hatching at 72 hpf and the completion of organogenesis at 120 hpf, all genes except *mate4* reached moderately high to high levels of expression (Fig. 3A).

The complex expression pattern of the *mate* transcripts in zebrafish tissues is shown in the Fig. 3B heat map. The highest expression was found in the kidneys, with *mate3* (in males) and *mate7* (in both genders) present at very high expression levels, followed by *mate6* and *mate8* (in both genders) with high expression, and *mate5* (in both genders) and *mate4* (in males) with moderate expression. *Mate3* showed pronounced gender differences in the kidney, with approximately 1,500-fold higher expression in males.

In liver, *mate6* and *mate8* were expressed at moderately high expression levels, followed by moderate levels of *mate7* in males. *Mate7* showed notably higher expression in male liver (20 times). *Mates* generally showed lower expression in the gills than in other tissues. All genes were moderately expressed except for the low expression of *mate6* and *mate5* in females. Sex differences were observed for *mate5* and *mate7*, which showed 10 and 2 times greater expression in males, respectively. In the intestine, the predominant *mate* genes are *mate6* in males (high expression) and *mate3* and *mate5* in males (moderately high expression). In females, all genes were expressed at low to moderate levels. In the testes, *mates* were generally highly expressed: *mate7* showed very high expression levels, followed by high expression of *mate3, mate4, mate5* and *mate8* and moderate expression of *mate6*. By contrast, in the ovaries, all genes showed low to moderate expression levels. In the zebrafish brain, *mate7* and *mate8* were highly expressed, as was *mate6* in males, followed by moderately high expression of *mate3* in males. Gender differences in *mate* expression in the brain were found for *mate3* and *mate6*, which showed 6 times higher expression in males. In the eye, *mates* were generally highly expressed, with high expression levels of *mate4* in females and *mate7* in both genders, followed by moderately high expression of *mate6* and *mate8* in both genders and *mate5* in females.

### Functional characterization

**Figure 4:**
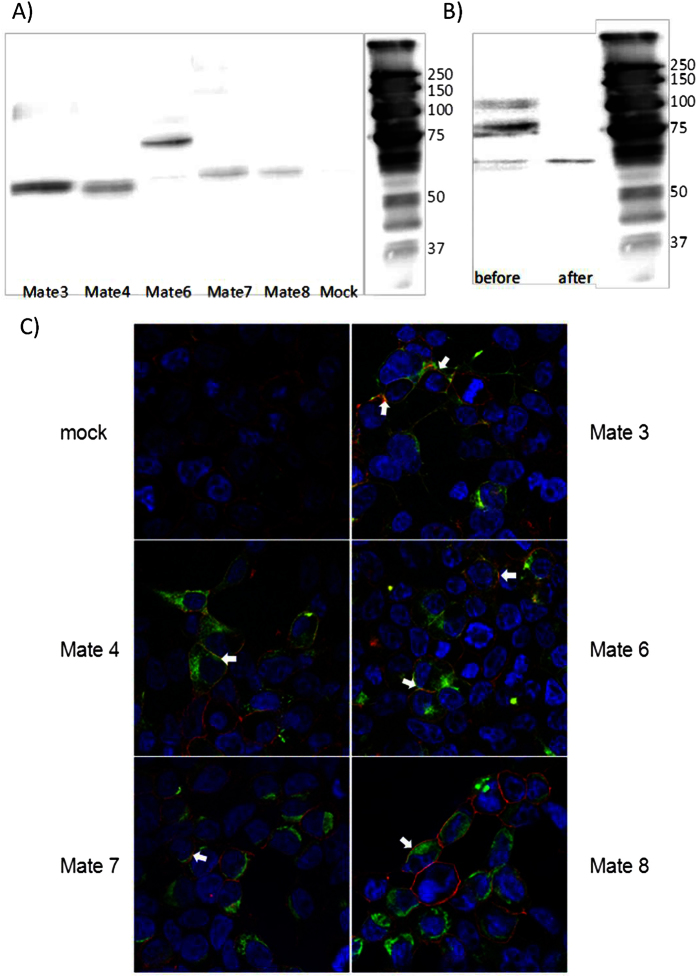
Expression and localization of zebrafish Mate transporters in transiently transfected HEK293T cells. (**A**) Western blot analysis of the total cell lysate (TCL) of HEK293T/Mate overexpressing cells. Zebrafish Mates are present as approx. 65 kDa monomers. Western blots were performed in the presence of reducing agent dithiothreitol (DTT) with anti-His antibody, and visualized with chemiluminescence. (**B**) Western blot analysis of the total cell lysate (TCL) of HEK293T/Mate6 overexpressing cells before and after deglycosylation. TCL of HEK293T/Mate6 overexpressing cells was treated with PNGase F. TCL before and after treatment is shown. **(C**) Cell localization of Mate3, Mate4, Mate6, Mate7 and Mate8 after overexpression in HEK293T cells. Immunocytochemistry was performed with fluorescein conjugated secondary antibody (FITC) that binds to the primary Xpress antibody and stains the protein in green. Nuclei are dyed in blue with DAPI (4′, 6-diamidino-2-phenylindole), and plasma membranes are stained in red after binding of primary antibody Na, K-ATPase and Cy3-conjugated IgG secondary antibody (all anti-mouse). White arrows indicate examples where protein is present in the cell membrane. The color turns to orange because of the overlap of green and red, or it remains light green when overexpression of protein is dominant over red stained membranes. Cytosolic forms are seen as green areas in the cytoplasm.

Transport kinetics were determined for five zebrafish Mate proteins heterologously overexpressed in HEK293T cells: Mate3, 4 and 6–8 (Table 1). Mate5 was not characterized due to the problems with plasmid propagation (see the Methods section). All other zebrafish Mates were successfully expressed and correctly localized in the cytoplasmic membrane in transiently transfected HEK293T cells (Fig. 4C). The molecular weight of Mate3, 4, 7 and 8 is approximately 65 kDa, as shown in the Western blot analysis (Fig. 4A), which corresponds to the molecular weight predicted from the amino acid sequences of each protein. However, in the case of Mate6, the dominant protein band was approximately 75 kDa. This corresponds to the glycosylated form of the transporter, as deglycosylation treatment with the PNGase F enzyme produced one major band of approximately 65 kDa, which is the molecular weight of Mate6 predicted from the amino acid sequence (Fig. 4B).

All zebrafish Mates transported the model cationic dye 4′, 6-diamidino-2-phenylindole (DAPI) with high affinity, as well as the model cation 4-(4-(dimethylamino)styryl)-N-methylpyridinium iodide (ASP^+^) with moderate to high affinity (Table 1). Four additional model cationic compounds were tested: rhodamine 123 (Rh123) and ethidium bromide (EtBr) were transported by all 5 zebrafish Mates, whereas amiloride and berberine were substrates of Mate3, 6 and 7 but were not transported by Mate4 and 8 (Table 1). All relevant concentration-response curves and DAPI time-response curves for the selected concentrations are shown in the Supplemental material (Supplemental Figs S3A–E and S4).

Mate3 showed a high affinity toward DAPI and EtBr: the *K*_*m*_ values were 2.83 ± 0.34 μM and 5.56 ± 0.50 μM, respectively, with similar affinities toward ASP^+^, Rh123 and berberine (*K*_*m*_ of 13.4 ± 1.55 μM, 8.52 ± 1.25 μM and 14.4 ± 5.14 μM, respectively) and moderate affinity toward amiloride (*K*_*m*_ = 33.4 ± 4.57 μM) (Table 1, Supplemental Fig. S3A). The transport rate was the highest for EtBr, followed by DAPI, ASP^+^, berberine and Rh123, with the lowest transport rate for amiloride (Table 1). Mate4 showed a very high affinity, in the nanomolar range, toward DAPI, Rh123 and EtBr (*K*_*m*_ of 0.29 ± 0.04 μM, 0.32 ± 0.03 μM and 0.45 ± 0.08 μM, respectively), followed by a high affinity toward ASP^+^ (*K*_*m*_ = 4.49 ± 0.63 μM). Amiloride and berberine were not substrates for Mate4 (Table 1, Supplemental Fig. S3B). The transport rate was moderate for DAPI, ASP^+^ and EtBr and very low for Rh123 (Table 1). Mate6 showed a very high affinity toward DAPI (*K*_*m*_ = 0.26 ± 0.05 μM), followed by a high affinity toward Rh123 (*K*_*m*_ = 1.73 ± 0.16 μM), EtBr (*K*_*m*_ = 2.98 ± 0.18 μM), ASP^+^ (*K*_*m*_ = 6.23 ± 1.02 μM), berberine (*K*_*m*_ = 8.35 ± 1.0 μM) and amiloride (*K*_*m*_ = 11.1 ± 2.12 μM) (Table 1, Supplemental Fig. S3C). The transport rate was high for EtBr, berberine and ASP^+^, followed by a moderate rate of transport for DAPI, Rh123 and amiloride (Table 1). Mate7 showed a very high affinity toward Rh123 and EtBr (*K*_*m*_ of 0.22 ± 0.03 μM, and 0.79 ± 0.19 μM, respectively) and a high affinity toward DAPI, berberine, ASP^+^ and amiloride (*K*_*m*_ of 1.44 ± 0.19 μM, 1.76 ± 0.38 μM, 3.51 ± 0.27 μM and 20.6 ± 4.20 μM, respectively). The transport rates for all cationic dyes were in the low to moderate range (*V*_*m*_ = 4.79–52.3 pmol/mg protein/min), except in the case of DAPI, which showed a high transport rate (*V*_*m*_ = 193 ± 9.15 pmol/mg protein/min) (Table 1, Supplemental Fig. S3D). Mate8 showed a very high affinity toward DAPI, Rh123 and EtBr (*K*_*m*_ of 0.55 ± 0.07 μM, 0.88 ± 0.14 μM and 0.85 ± 0.12 μM, respectively) and a high affinity toward ASP^+^ (*K*_*m*_ = 2.34 ± 0.37 μM), whereas amiloride and berberine were not transported (Table 1). Transport rates for all 4 dyes were in the lower range (*V*_*m*_ = 12.3–24.4 pmol/mg protein/min) (Table 1, Supplemental Fig. S3E).

**Figure 5:**
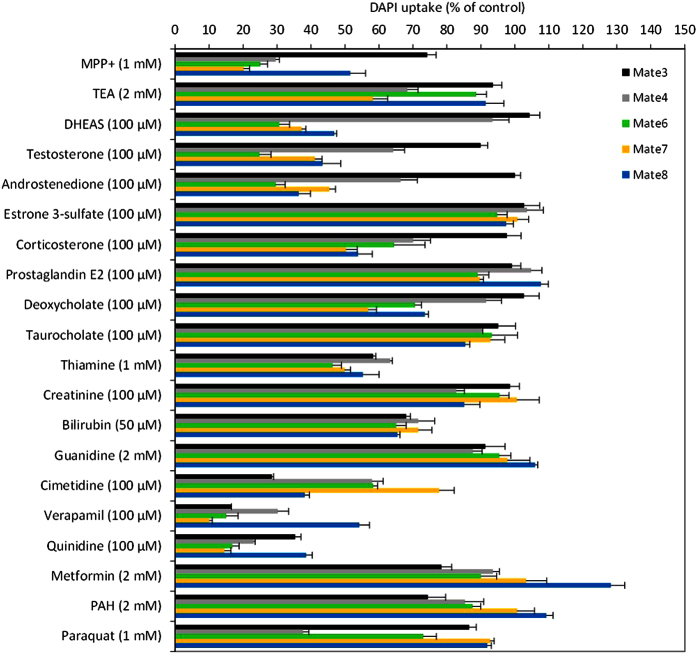
Interaction of zebrafish Mate3, Mate4 and Mate6–8 with selected interactors of mammalian MATEs/Mates. Data are expressed as a percentage (%) of 4′, 6-diamidino-2-phenylindole (DAPI) uptake kinetics after co-incubation with each modulator (50 μM–2 mM) relative to DAPI uptake kinetics in the absence of a modulator, which is set to 100%. Data represent mean ± SE from three to five independent experiments (n = 3–5).

Following optimization of the transport activity assays, we tested a series of known interactors of mammalian MATEs/Mates, including the model cationic substrates 1-methyl-4-phenylpyridinium (MPP^+^) and TEA, potential physiological interactors (steroid hormones, thiamine, creatinine, guanidine, bile salts and bilirubin) and xenobiotic substrates (mostly pharmaceutical compounds known to be transported by mammalian MATEs, e.g., cimetidine and metformin) (Fig. 5). This type of transport assay does not distinguish between an interactor that is transported by the protein and one that is a non-competitive inhibitor. It does, however, demonstrate whether a compound interferes (or not) with the transport of a model fluorescence substrate. Among the six cationic dyes listed above, we chose DAPI for further functional analysis due to the high to very high affinity of all five zebrafish Mates toward DAPI, the moderate to high transport rates (Table 1), and highly processive nature of the DAPI uptake assay (i.e., it is possible to measure uptake in real time).

The model cationic substrates MPP^+^ and TEA showed either very weak interaction (IC_50_ = 2430.5 μM) or did not interact with DAPI uptake mediated by zebrafish Mate3. Similarly, zebrafish Mate3 did not show notable interaction with physiological interactors (Fig. 5), except for a weak interaction with testosterone (IC_50_ = 668.8 μM) and a very weak interaction with thiamine (IC_50_ = 2021.5 μM) (Table 2, Supplemental Fig. S5D and H). The xenobiotic compounds cimetidine, verapamil and quinidine inhibited zebrafish Mate3-mediated DAPI uptake with an IC_50_ of 30.3, 12.3 and 58.4 μM, respectively (Table 2, Supplemental Fig. S5I–K). Zebrafish Mate4 showed weak and very weak interactions with both the model cations MPP^+^ and TEA and various physiological interactors (DHEAS, testosterone, androstenedione, corticosterone and thiamine), respectively (Table 2, Supplemental Fig. S2A–H). Quinidine and verapamil were strong and moderate inhibitors of DAPI uptake (IC_50_ of 20.9 and 32.5 μM, respectively), whereas cimetidine and paraquat showed weak inhibition (IC_50_ of 133.8 and 462.5 μM, respectively) (Table 2). Mate6 strongly interacted with MPP^+^ (IC_50_ = 27.9 μM), the steroid hormone androstenedione (IC_50_ = 25.3 μM), and the xenobiotics verapamil (IC_50_ = 4.1 μM) and quinidine (IC_50_ = 6.2 μM), while it showed a moderate interaction with testosterone, DHEAS and corticosterone (Table 2). Mate7 showed moderate and very weak interactions with MPP^+^ and TEA (IC_50_ of 93.6 and 3311.3 μM, respectively). Androstenedione, verapamil and quinidine showed a strong interaction with Mate7, followed by testosterone and DHEAS, with moderate interaction strength. Corticosterone, deoxycholate, cimetidine and thiamine weakly interacted with Mate7, while the paraquat interaction was too weak to determine the IC_50_ within its solubility range (Table 2). Mate8-mediated DAPI uptake was not inhibited by TEA (Fig. 5), whereas it weakly interacted with MPP^+^ (Table 2). Androstenedione, DHEAS, corticosterone, cimetidine and quinidine were strong interactors of Mate8, while testosterone and verapamil showed a moderate interaction, followed by deoxycholate, a weak interactor, and thiamine and paraquat, which were very weak interactors of zebrafish Mate8 (Table 2).

## Discussion

MATE/Mate transporters have recently been recognized as important components of the phase III cellular detoxification in humans, mice and rats^7^,^15^. However, knowledge of the non-mammalian Mate proteins was lacking. Therefore, we focused on elucidating their identity, gene expression, and transport properties to obtain the first comprehensive data set to help determine the possible roles of these transporters in zebrafish, an important model vertebrate species.

We identified six *mate* genes in the zebrafish genome and found that fish Mate proteins clearly form a distinct cluster from tetrapod MATE/Mates (Fig. 1). Based on the phylogenetic analysis, direct orthologous relationships could not be determined among fish and mammalian MATE/Mate proteins. To address this obstacle, we performed synteny analysis which showed that zebrafish *mates* are syntenic to human *MATE* genes (Fig. 2). The observed differences in the surrounding gene environments are most likely a consequence of the teleost-specific whole genome duplication (WGD) that has been shown to form double conserved synteny blocks (DCS)^34^. DCS are defined^35^,^36^ as gene regions in non-duplicated species that are found on two different chromosomes in the species that underwent the WGD, although the genes may not be adjacent in the duplicated species. Therefore, considering the similar gene environments among the two zebrafish *mate* clusters, i.e., *mate3, 4* and *6* on chromosome 21 and *mate5, 7* and *8* on chromosome 15 (Fig. 2), we propose that these two zebrafish clusters arose from a genome duplication event.

The first indication of the potential importance of Mates was obtained by gene expression analyses in both zebrafish embryos and adults. Determination of the expression of the *mate* genes in zebrafish embryos at early developmental stages (Fig. 3A) revealed several important insights. First, all of the *mate* transcripts were constitutively and differentially expressed during embryonic development. The initial stages of development (1 to 4 hpf) were characterized by low expression of all *mates*, likely as a consequence of maternal transfer, followed by gradual increases in expression in the later stages and stabilization of expression between the finish of hatching at 72 hpf and completion of organogenesis at 120 hpf. Second, high expression of *mate6* and *mate7* at the onset of embryonic transcriptional activity at 6 hpf suggests that these proteins may have specific roles in embryonic development. Finally, expression levels in the larvae (72–120 hpf) correlate well with the expression levels in the adult tissues (Fig. 3B).

All six zebrafish *mates* are ubiquitously expressed in the analyzed tissues of adult zebrafish. Unlike human, mouse and rat *MATEs/mates*, none of the six zebrafish genes predominates in a single tissue. Human MATE1 is present in several tissues and is the predominant MATE in kidney and liver. It is localized in the apical membrane of the proximal tubule cells and hepatocytes, where it mediates the efflux of compounds into urine and/or bile^37^. Mouse Mate1a and b isoforms have similar tissue distribution patterns with dominant presence in kidney. Mate1a is also present in the liver^7^,^9^,^11^, while rat Mate1 is found in the kidney and placenta^4^,^12^,^38^. Human MATE2 (and its variant MATE2-K) is predominantly expressed in the apical membrane of the proximal renal tubules, whereas mouse and rat Mate2 are predominantly found in the testes^10^ and have not been notably detected in other tissues^37^. In comparison, similar to mammalian *MATEs/mates*^37^ transcripts, the highest expression of zebrafish *mates* is found in the kidney and testes and to a lesser extent in the liver and brain (Fig. 3B). However, apart from these general similarities, the tissue expression pattern of each zebrafish gene cannot be directly matched to a human, mouse and/or rat *MATE/mate* transporter(s). Among zebrafish *mates, mate6, 7* and *8* are the only ones present in both the kidney and liver, with moderate to very high expression levels in both genders, whereas *mate4* and *5* are predominantly expressed in the testes and kidney and are not present in the liver at notable levels (Fig. 3B). In that sense, *mate4* and *mate5* resemble human MATE2, which is also not found in the liver, and mouse and rat Mate2, which are found in the testes at high expression levels. Summarizing our tissue gene expression data and considering the high renal expression levels, we propose that all six zebrafish Mates are important transporters in fish kidney, and Mate6 and Mate8 may be important in the liver, where they are found at moderate levels (Fig. 3B). In the testes, with the exception of *mate6*, all *mates* are highly expressed, indicating an important transport function in this tissue, possibly in testosterone transport as proposed previously^10^ for human MATEs.

Following the results of our phylogenetic and gene expression analyses, we developed functional assays to identify Mate interactors (i.e., substrates and/or inhibitors) to elucidate the possible functional roles of zebrafish Mate transporters. We showed that six dyes—ASP^+^, DAPI, Rh123, berberine, amiloride and EtBr—are substrates of zebrafish Mate3, 6 and 7, and DAPI, ASP^+^, Rh123 and EtBr are also substrates of Mate4 and Mate8 (Table 1). Comparing the kinetic parameters of all zebrafish Mates for all the tested dyes, DAPI appears to have been the best choice for further functional analysis because all zebrafish Mates had high affinity toward this dye, as well as comparatively high maximal transport rates (Table 1). Therefore, we proceeded with the initial functional characterization of zebrafish Mates using DAPI as a model substrate.

The organic cations MPP^+^ and TEA are considered model substrates for mammalian MATE/Mates, and radiolabeled [^3^H]MPP^+^ and [^14^C]TEA are often used in inhibition assays to identify interactors of mammalian MATE/Mate transporters^3^. Consequently, we were able to compare the affinities of mammalian MATE/Mates toward MPP^+^ and TEA with the related IC_50_ values determined for zebrafish Mates (Table 2). The interaction of zebrafish Mate4, 6 and 7 with MPP^+^ (Table 2) is comparable to the affinity of human MATE1 toward MPP^+^ (*K*_*m*_ = 100 μM)^18^, while Mate3, 4 and 8 have lower affinities (Table 2). Unlike mammalian MATEs/Mates (human MATE1 and MATE2, rat and mouse Mate1), which do transport TEA^3^, zebrafish Mate3, 6 and 8 did not interact with TEA (Table 2). By contrast, zebrafish Mate7 interacted with TEA (Table 2), although at lower affinity than those reported for human MATE1 and MATE2 and rat and mouse Mate1 (*K*_*m*_ of 220–580, 375–1390, 260–570 and 410–890 μM respectively)^3^.

Following standardization of the DAPI transport activity assays, we selected and tested a set of known physiological interactors of mammalian MATEs/Mates, including steroid hormones and their conjugates, bile salts, thiamine, creatinine, bilirubin, and guanidine. Testosterone inhibited the DAPI transport of zebrafish Mate3, 4, 6, 7 and 8 at 100 μM (the interaction with Mate3 was very weak), similar to the interaction of human and murine MATE1/Mate1 and MATE2/Mate2^9^,^10^,^39^. Corticosterone, a known inhibitor of mammalian MATEs/Mates^10^,^12^,^32^ interacted with zebrafish Mate4, 6, 7 and 8 but not Mate3 (Table 2). DHEAS has been shown not to be transported by human MATEs, while information on its inhibitory effect is missing^18^. We found that DHEAS is a moderate to strong interactor of zebrafish Mate6, 7 and 8. Androstenedione strongly interacted with zebrafish Mate6, 7 and 8 (Fig. 5 and Table 2), which is comparable to results obtained for human and murine MATE1/Mate1^9^. Thiamine, a high-affinity substrate of human MATE1 and 2 (*K*_*m*_ of 3.5 μM and 3.9 μM, respectively)^40^, interacted with zebrafish Mates at variable interaction strengths (Fig. 5 and Table 2), while creatinine, a known substrate of human MATE1 and MATE2, did not interact with zebrafish Mates (Fig. 5). Data on the interaction of mammalian MATEs/Mates with deoxycholate are not currently available.

To assess a possible role in cellular defense for zebrafish and mammalian MATEs/Mates, two typical xenobiotic MATE/Mate substrates were tested: cimetidine and metformin. DAPI transport of zebrafish Mate3, 4, 6, 7 and 8 was reduced by cimetidine with IC_50_ values (Table 2) in the range of human MATE 1 and MATE2, as well as rat Mate1 (*K*_*m*_ of 7–170, 18–370, and 3 μM, respectively)^3^. However, the interaction with metformin substantially differed: the reported affinity of MATE1 toward metformin was in the range of 227–780 μM and the affinity of MATE2-K was 362–1980 μM, whereas zebrafish Mate3, 4, 6, 7 and 8 did not interact with metformin (Fig. 5). In addition, results obtained for other known interactors of mammalian MATEs/Mates, verapamil and quinidine, revealed moderate to strong interaction of both compounds with all zebrafish Mates (IC_50_ between 3.1 and 58.4 μM), with an affinity comparable to human MATE1 and MATE2-K (IC_50_ between 23 and 32 μM)^41^. Therefore, our functional characterization revealed that zebrafish and mammalian MATEs/Mates have both notable similarities and differences with respect to interaction strengths and profiles of their chemical interactors. They show similar interaction profiles for MPP^+^, cimetidine, steroid hormones, verapamil and quinidine, and they differ in the interactions with TEA, thiamine, metformin, and creatinine. Furthermore, zebrafish Mates interact with both endogenous and xenobiotic compounds (Fig. 5 and Table 2).

Based on the performed expression studies, transport activity assays and interaction profiling, herewith we propose several hypotheses on possible role of Mates in zebrafish. In embryos, considering high expression of Mate6 and 7, with peaks in gastrula (5–10 hpf) and segmentation period of development (10–24 hpf), we suggest two possible roles. The first one is defensive role through protection of early embryos against adverse environmental impacts. The excretion of toxic compounds from the cell or embryo is mediated by two transporter families: ATP-binding cassette (ABC) proteins and MATE transporters. The first evidence on defensive role of ABC transporters in early embryos was provided in 2004 by Hamdoun and colleagues^42^. Using the sea urchin embryo model they showed that pharmacological inhibition of the corresponding efflux activity sensitized embryos to the toxic compound vinblastine, implying that one role for the efflux transport activity is embryo protection from xenobiotics. Likewise, follow-up studies reveal that polyspecific membrane transporters are required for signaling, homeostasis, and protection of the embryo^43^. In zebrafish, Fischer *et al*.^44^ have shown the presence of ABCB4 P-glycoprotein-like efflux transporter in embryo that acts as an efflux pump keeping concentrations of the toxic compounds in embryos low. Therefore, high expression of zebrafish Mates as demonstrated in this study suggests for the first time potential role of Mate transporters in protection of early embryos.

Considering possible physiological function, our data clearly show potent interaction of zebrafish Mates with steroid hormones. However, the impact of steroid hormones on zebrafish embryogenesis is poorly understood, although some basic features of hormone synthesis are described. There is evidence that steroid hormones (cortisol and estradiol) are maternally provided to the zygote^45^ and that zebrafish, although biosynthesis pathway becomes active only from 48 hpf onwards, responds to the hormones before that point due to the presence of hormones of maternal origin^46^. In addition, the presence of maternal (decreasing from 0–6 hpf) and embryonic (increasing from 6–48 hpf) steroid receptor mRNA for estrogen, androgen and glucocorticoid receptors has also been shown^47^.

As to the role of zebrafish Mates in adults, considering the similarly high MATE/Mate expression in the kidney and liver, we propose that zebrafish Mate transporters play important roles in the excretion of xenobiotic and endogenous compounds through the kidney and liver as the main excretory pathways, similarly to mammalian MATEs/Mates. Along with the proposed cellular defense functions, our results suggest a possible role of specific zebrafish Mates in steroid transport and protection of the blood-testes barrier. In human testes, MATE1 expression is very low^8^, while both murine Mates and rat Mate2 are expressed in Leydig cells^9^,^10^,^48^, where they may be involved in the export of testosterone to the blood, as hypothesized by Hiasa *et al*.^10^ In addition, it has been shown^48^ that murine Mate2 is also highly expressed in Sertoli cells, which support and protect spermatogenic Leydig cells, acting as a blood-testis barrier. Ovarian Mates showed low expression in mice^48^, similar to zebrafish. Therefore, considering the high expression of zebrafish Mate7 and Mate8 in the testes and their interaction with DHEAS, testosterone and androstenedione (Table 2), Mate7 and Mate8 might be involved in the transport of steroids and xenobiotics through the blood-testes barrier.

Several transporters in the mammalian brain are involved in hormone export or restriction of chemical accumulation in the blood-brain barrier^16^. Mates present in the zebrafish brain, however, show higher gene expression than their human counterparts. Considering the high expression of zebrafish Mate6, 7 and 8 in the brain (Fig. 3B) and the preference toward transport of cations, zebrafish Mates might be involved in the transport of neurotransmitters across the BBB, similar to the cationic SLC transporters from the SLC22 family^49^.

Data from the available studies revealed very low expression levels of mammalian MATEs/Mates in the intestine^8^,^12^,^48^. Nevertheless, moderately high to high expression of zebrafish Mate3, 5 and 6 in male fish indicates a role in the elimination of physiological metabolites and/or xenobiotics via efflux back into the intestinal lumen, similar to the ABC transporters^50^.

In conclusion, this study reveals novel insights into the evolution of the MATE/Mate transporter family and provides the first comprehensive data set on Mate transporters in zebrafish, an important vertebrate model species. A sound methodological basis for future studies investigating the Mate interactors is presented. Finally, plausible hypotheses related to the possible roles of Mate proteins in the transport of physiological and/or xenobiotic substrates in zebrafish embryos and/or adults are proposed. They remain to be more specifically addressed in follow-up studies through detailed functional characterization of single transporter/s in suitable expression systems and zebrafish functional genomics studies using targeted gene knockdown.

## Additional Information

**How to cite this article**: Lončar, J. *et al*. The first characterization of multidrug and toxin extrusion (MATE/SLC47) proteins in zebrafish (*Danio rerio*). *Sci. Rep.* **6**, 28937; doi: 10.1038/srep28937 (2016).

## Supplementary Material

Supplementary Information

## Figures and Tables

**Table 1:** Kinetic parameters, *K*_*m*_ (μM) and *V*_*max*_ (pmol/mg protein/min) of the uptake of fluorescent dyes.

	Mate3		Mate4		Mate6		Mate7		Mate8	
	*K*_*m*_	*v*_*max*_	*K*_*m*_	*v*_*max*_	*K*_*m*_	*v*_*max*_	*K*_*m*_	*v*_*max*_	*K*_*m*_	*v*_*max*_
DAPI	2.83 ± 0.34	436 ± 21.8	0.29 ± 0.04	47.7 ± 2.27	0.26 ± 0.05	44.3 ± 1.80	1.44 ± 0.19	193 ± 9.15	0.55 ± 0.07	24.4 ± 0.70
ASP^+^	13.4 ± 1.55	296 ± 14.0	4.49 ± 0.63	23.6 ± 1.23	6.23 ± 1.02	160 ± 10.8	3.51 ± 0.27	52.3 ± 1.26	2.34 ± 0.37	22.2 ± 1.09
Rh123	8.52 ± 1.25	105 ± 5.16	0.32 ± 0.03	1.03 ± 0.03	1.73 ± 0.16	55.6 ± 1.48	0.22 ± 0.03	4.79 ± 0.17	0.88 ± 0.14	12.3 ± 0.61
EtBr	5.56 ± 0.50	825 ± 25.5	0.45 ± 0.08	37.4 ± 2.02	2.98 ± 0.18	504 ± 9.79	0.79 ± 0.19	26.3 ± 1.82	0.85 ± 0.12	17.0 ± 0.59
Amiloride	33.4 ± 4.57	53.1 ± 4.0	NT	NT	11.1 ± 2.12	24.8 ± 1.54	20.6 ± 4.20	19.8 ± 1.84	NT	NT
Berberine	14.4 ± 5.14	242 ± 46.9	NT	NT	8.35 ± 1.00	215 ± 9.72	1.76 ± 0.38	28.0 ± 2.39	NT	NT

4-(4-(dimethylamino)styryl)-N-methylpyridinium iodide (ASP^+^), 4′,6-diamidino-2-phenylindole (DAPI), rhodamine 123 (Rh123), ethidium bromide (EtBr), berberine and amiloride for zebrafish Mates (Mate3, 4, 6, 7 and 8). Each value represents mean ± SD from triplicate determinations of a representative experiment. NT – no transport found. Dose response graphs for each dye are shown in Fig. S3A–E.

**Table 2:** IC_50_ values for a set of chosen zebrafish interactors for Mate3, Mate4 and Mate6–8 determined using the 4′, 6-diamidino-2-phenylindole (DAPI) inhibition assay.

Compound	Mate3		Mate4		Mate6		Mate7		Mate8	
	IC_50_	SE	IC_50_	SE	IC_50_	SE	IC_50_	SE	IC_50_	SE
MPP^+^	2430.5	59.2	434.3	7.1	27.9	0.8	93.6	1.4	977.9	102.3
TEA	NE	–	5244.5	208.7	NE	–	3311.3	325.7	NE	–
DHEAS	NE	–	313.4	38.5	39.1	3.7	60.2	2.3	26.9	1.1
Testosterone	668.8	44.1	651.2	43.2	39.0	1.6	42.0	2.5	50.5	3.8
Androstenedione	NE	–	114.2	13.6	25.3	0.4	14.9	1.5	20.1	4.1
Corticosterone	NE	–	237.8	9.4	99.3	14.8	99.6	8.2	22.1	3.8
Deoxycholate	NE	–	NE	–	NE	–	202.9	9.0	274.4	40.8
Thiamine	2021.5	21.6	805.0	22.9	724.5	100.3	935.2	39.8	1896.0	100.5
Cimetidine	30.3	2.5	133.8	10.7	357.1	35.6	572.7	14.9	4.5	0.2
Verapamil	12.3	1.1	32.5	3.1	4.1	0.5	6.4	0.3	51.5	3.2
Quinidine	58.4	3.0	20.9	2.3	6.2	0.7	10.0	0.1	3.1	0.9
Paraquat	NE	–	462.5	12.9	5729.5	658.5	NE	–	6294.0	769.6

IC_50_ values given in μM were determined by fitting data to sigmoidal four parameters dose–response model (variable slope) in the GraphPad Prism 5. IC_50_ values and standard errors (SE) were calculated from three independent experiments (n = 3). NE – no effect in the compound solubility range; MPP^+^ is 1-methyl-4-phenylpiridinium; TEA is tetraethylammonium; DHEAS is dehydroepiandrosterone sulfate. Representative dose response curves for each compound are shown in Fig. S5A–L.
