# Supplementary material for: The first characterization of multidrug and toxin extrusion (MATE/SLC47) proteins in zebrafish (Danio rerio)
Source: Sci Rep. 2016 Jun 30;6:28937. doi: 10.1038/srep28937 (PMC4928094; doi:10.1038/srep28937)
Supplement: Supplementary Information [file srep28937-s1.pdf]

Supplementary material

The first characterization of multidrug and toxin extrusion (MATE/SLC47) proteins in zebrafish (*Danio rerio*)

Jovica Lončar[1]ǂ, Marta Popović[1, 3]ǂ, Petra Krznar[4], Roko Zaja[1, 2], Tvrtko Smital[1]*

[1]*Laboratory for Molecular Ecotoxicology, Division for Marine and Environmental Research, Ruđer Bošković Institute, Zagreb, Croatia*

[2]*Sir William Dunn School of Pathology, University of Oxford, Oxford, England, United Kingdom*

[3]*Oxford Institute for Radiation Oncology, University of Oxford, Oxford, England, United Kingdom*

[4]*Institute of Molecular Systems Biology, ETH, Zurich, Switzerland*

* Corresponding author: smital@irb.hr

ǂ These authors contributed equally to this work.

Table S1. Primer sequences used for relative quantification of zebrafish *mates* using qRT-PCR analysis.

Protein name	Primer sequence 5→3	Final conc.(nM)	Slope	Intercept	Efficiency[%]
DrMate3	F TCGGATGTACCAAAGATGT	300	-3.69	35.1	86.7
	R CGGTAATGCCCGCTATGG	300			
DrMate4	F GCAGCACAGGAGAACACTGA	300	-3.23	34.6	104
	R CCAACTGTGACCAATGCAAC	300			
DrMate5	F GGCTGTGATGTGAATGATGC	300	-3.04	27.4	113
	R GGCGCAGCACTAAAACACTC	300			
DrMate6	F TCACGCTGTACGTTTTCCTG	300	-3.35	31.6	98.8
	R ACACTGCTGCAATCTTCTGC	300			
DrMate7	F CTCAGGATCAGGACGAGAGG	300	-3.22	26.4	105
	R CTGAGTGGTGGACAGCAGAA	300			
DrMate8	F TCTGCAATGACAGCTCCTGATAGTAT	300	-3.44	38.8	95.3
	R GGCTCCACGTTCGGATCG	300			
EF1α	F CCTGGGAGTGAAACAGCTGATC	300	−3.43	42.5	95.5
	R GCTGACTTCCTTGGTGATTTCC	300			

Table S2. Primers used for cloning of zebrafish *mates*. Primer sequences, restriction enzyme sites introduced in the primer sequences (bold letters), and T_m values are shown.

Gene name	Primer sequence 5 -> 3	Restriction enzymes	Tm [°C]
Mate3	F TTA**GCTAGCGAATTC**ATGGACAGTATTACCAGTTACAA	NheI-EcoRI	57
	R TTA**GCGGCCGCGGTACC**TCATCTAAGATATCTAACAAGCA	NotI-KpnI	55
Mate4	F TTA**GCTAGCGAATTC**ATGGAAATGAACAGTATTCATAC	NheI-EcoRI	57
	R TTA**GCGGCCGCGGTACC**TCAAATAACAAATCTAATCAGCA	NotI-KpnI	58
Mate5	F TTA**GCTAGCGAATTC**ATGAACGAAAGAATGAACGAAG	NheI-EcoRI	62
	R TTA**GCGGCCGCGGTACC**TCATCTTTTAAGACTGTACTTGA	NotI-KpnI	55
Mate6	F TTA**GCTAGCGAATTC**ATGCCTGAGGCTGGCG	NheI-EcoRI	67
	R TTA**GGTACCTCTAGA**TTAGCTCATTGTAATATTGAGAA	KpnI-XbaI	55
Mate7	F TTA**AAGCTTGAATTC**ATGGACGTCTCCAGTCCTGG	HindIII-EcoRI	67
	R TTA**GCGGCCGCCCGGG**TCAAATGAAACTCGTTGAATAGA	NotI-SmaI	61
Mate8	F TTA**GCTAGCGAATTC**ATGGACGTTTCAAGTCCCAC	NheI-EcoRI	64
	R TTA**GGTACCTCTAGA**TTATAATGACGCAGAAGTGGAAT	KpnI-XbaI	62

Table S3. Protein annotation and accession numbers of protein sequences used in the phylogenetic analysis of MATE superfamily (Multidrug Extrusion; gene name *SLC47A*) in metazoa. Species abbreviations: Hs, *Homo sapiens*; Mm, *Mus musculus*; Rn, *Rattus norvegicus*; Gg, *Gallus gallus*; Tg, *Taeniopygia guttata*; Xt, *Xenopus tropicalis*; Ac, *Anolis carolinensis*; Lc, *Latimeria chalumnae*; Dr, *Danio rerio*; Ga, *Gasterosteus aculeatus*; Gm, *Gadus morhua*; Ol, *Oryzias latipes*; Tr, *Takifugu rubripes*; Bf, *Branchiostoma floridae*; Sk, *Saccoglossus kowalevskii*; Lg, *Lottia gigantea*; Ct, *Capitella teleta*; Nv, *Nematostella vectensis*; Aq, *Amphimedon queenslandica*; Ta, *Trichoplax adhaerens*; Mb, *Monosiga brevicollis*; Sc, *Saccharomyces cerevisiae*; Ec, *Escherichia coli*; Vc, *Vibrio cholera*. Protein sequences with accession numbers starting with 'NP', 'XP', 'YP' or 'WP'' were retrieved from NCBI database; the ones starting with 'ENS' or 'PAC' were retrieved from ENSEMBL database; and protein IDs in number format were retrieved from JGI database.

Protein annotation	Accession number	Length (aa)
HsMATE1	NP_060712.2	570
HsMATE2	NP_690872.2	602
HsMATE2-K	NP_001093116.1	566
HsMATE2-B	BAF37007.1	219
HsMATE2_tv3	NP_001243592.1	580
MmMate1a	AAH31436	532
MmMate1b	NP_080459.2	567
MmMate2	NP_001028714.1	573
RnMate1	NP_001014140.1	566
RnMate2	NP_001178849.1	572
GgMate1	NP_001129151.1	579
GgMate2	NP_001025891.2	475
TgMate1	XP_012424121.1	536
TgMate2	XP_012424122.1	622
AcMate1	XP_008123627.1	527
AcMate2	XP_003229800.2	373
XtMate1	NP_001096440.1	574
XtMate2	XP_012812968.1	600
XtMatelike1	XP_002934402.2	562
XtMatelike2	XP_002933880.2	550
XtMatelike3	XP_012813618.1	565
LcMate1	ENSLACP00000003119	575
LcMate2	ENSLACP00000001877	494
**DrMate3**	NP_001073648.1	590
**DrMate4**	NP_001274473.1	586
**DrMate5**	XP_005157715.1	548
**DrMate6**	NP_001014332.2	629
**DrMate7**	NP_001289183.1	607
**DrMate8**	XP_003201757.2	588
GaMate3	ENSGACP00000027262	566
GaMate4	ENSGACP00000024450	563
GaMate5	ENSGACP00000014451	497
GaMate6	ENSGACP00000027266	577
GaMate7	ENSGACP00000014470	530
GmMate3	ENSGMOP00000015086	454
GmMate5	ENSGMOP00000011176	458
GmMate6	ENSGMOP00000012623	283
OlMate4	ENSORLP00000002448	569
OlMate5	ENSORLP00000006032	566
OlMate6	ENSORLP00000006776	567
OlMate7	ENSORLP00000006011	440
OlMate8	ENSORLP00000005994	576
TrMate3	ENSTRUP00000002595	469
TrMate4	ENSTRUP00000011208	580
TrMate5	ENSTRUP00000007545	524
TrMate6a	ENSTRUP00000000290	570
TrMate6b	ENSTRUP00000003131	560
BfMatelike1	XP_002605941.1	715
BfMatelike2	183877	431
SkMatelike1	XP_002737051.1	647
SkMatelike2	XP_002737052.1	622
SkMatelike3	XP_006820737.1	553
LgMatelike1	XP_009051070.1	659
NvMatelike1	XP_001638537	408
CtMatelike1	164649	427
CtMatelike2	ELU15421.1	612
TaMatelike1	XP_002108992	533
ScMatelike1	NP_011897.1	581
ScMatelike2	NP_010625.1	695
AqMatelike1	XP_003383317.1	671
MbMatelike1	XP_001744290	446
EcNorM	YP_025307.1	457
VcNorM	AAF94694.1	461

Figure S1.

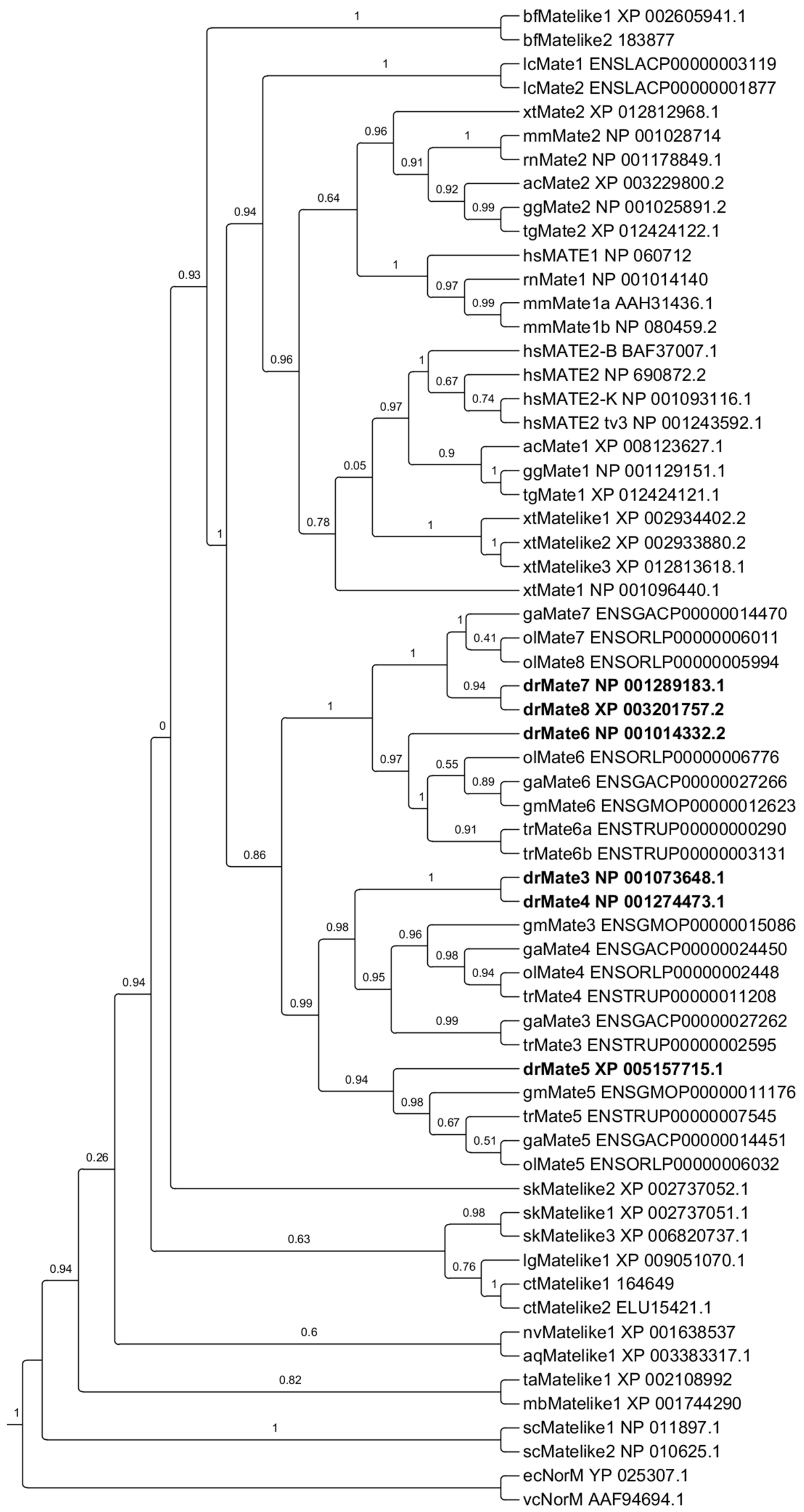
bfMatelike1 XP 002605941.1
bfMatelike2 183877
lcMate1 ENSLACP00000003119
lcMate2 ENSLACP00000001877
xtMate2 XP 012812968.1
mmMate2 NP 001028714
rnMate2 NP 001178849.1
acMate2 XP 003229800.2
ggMate2 NP 001025891.2
tgMate2 XP 012424122.1
hsMATE1 NP 060712
rnMate1 NP 001014140
mmMate1a AAH31436.1
mmMate1b NP 080459.2
hsMATE2-B BAF37007.1
hsMATE2 NP 690872.2
hsMATE2-K NP 001093116.1
hsMATE2 tv3 NP 001243592.1
acMate1 XP 008123627.1
ggMate1 NP 001129151.1
tgMate1 XP 012424121.1
xtMatelike1 XP 002934402.2
xtMatelike2 XP 002933880.2
xtMatelike3 XP 012813618.1
xtMate1 NP 001096440.1
gaMate7 ENSGACP00000014470
olMate7 ENSORLP00000006011
olMate8 ENSORLP00000005994
drMate7 NP 001289183.1
drMate8 XP 003201757.2
drMate6 NP 001014332.2
olMate6 ENSORLP00000006776
gaMate6 ENSGACP00000027266
gmMate6 ENSGMOP00000012623
trMate6a ENSTRUP00000000290
trMate6b ENSTRUP00000003131
drMate3 NP 001073648.1
drMate4 NP 001274473.1
gmMate3 ENSGMOP00000015086
gaMate4 ENSGACP00000024450
olMate4 ENSORLP00000002448
trMate4 ENSTRUP00000011208
gaMate3 ENSGACP00000027262
trMate3 ENSTRUP00000002595
drMate5 XP 005157715.1
gmMate5 ENSGMOP00000011176
trMate5 ENSTRUP00000007545
gaMate5 ENSGACP00000014451
olMate5 ENSORLP00000006032
skMatelike2 XP 002737052.1
skMatelike1 XP 002737051.1
skMatelike3 XP 006820737.1
lgMatelike1 XP 009051070.1
ctMatelike1 164649
ctMatelike2 ELU15421.1
nvMatelike1 XP 001638537
aqMatelike1 XP 003383317.1
taMatelike1 XP 002108992
mbMatelike1 XP 001744290
scMatelike1 NP 011897.1
scMatelike2 NP 010625.1
ecNorM YP 025307.1
vcNorM AAF94694.1

Figure S1. Phylogenetic analysis of MATE/Mate family (Multidrug and toxin extrusion). Phylogenetic tree was constructed with maximum likelihood method. Branch support is estimated with the approximate likelihood ratio test (aLRT). SLC22 gene family was used as an outgroup (not shown in the tree). Species abbreviations: Hs, Homo sapiens; Rn, *Rattus norvegicus*; Mm, *Mus musculus*; Gg, *Gallus gallus*; Tg, *Taeniopygia guttata*; Ac, *Anolis carolinensis*; Xt, *Xenopus tropicalis*; Dr, *Danio rerio*; Tn, *Tetraodon nigroviridis*; Tr, *Takifugu rubripes*; Ga, *Gasterosteus aculeatus*; Ol, *Oryzias latipes*; Gm, *Gadus morhua*; Lc, *Latimeria chalumnae*; Le, *Leucoraja erinacea*; Bf, *Branchiostoma floridae*; Pm, *Petromyzon marinus*; Od, *Oikopleura dioica*; Ci, *Ciona intestinalis*; Sk, *Saccoglossus kowalevskii*; Sp, *Strongylocentrotus purpuratus*; Ct, *Capitella teleta*; Nv, *Nematostella vectensis*; Aq, *Amphimedon queenslandica*; Ta, *Trichoplax adhaerens*; Mb, *Monosiga brevicollis*; Sc, *Saccharomyces cerevisiae*; Ec, *Escherichia coli*; Vc, *Vibrio cholera*.

Figure S2.

A. liver

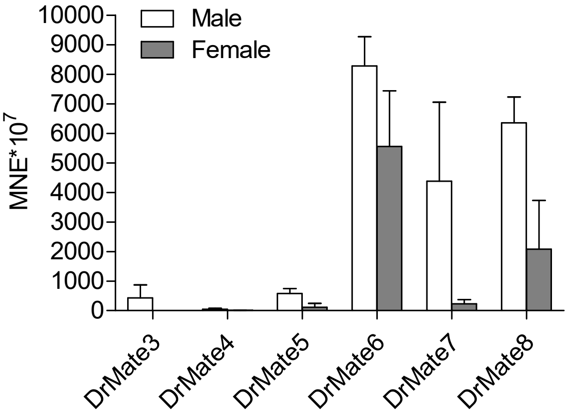

B. kidney

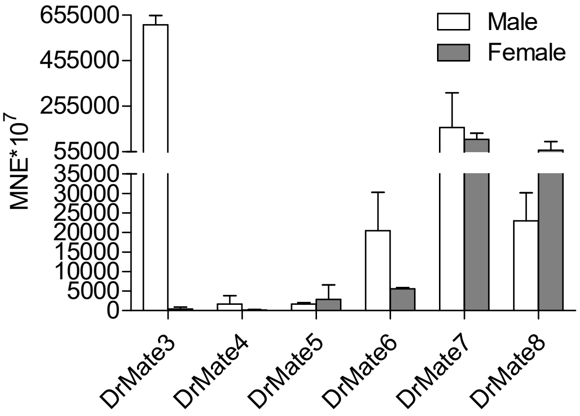

C. intestine

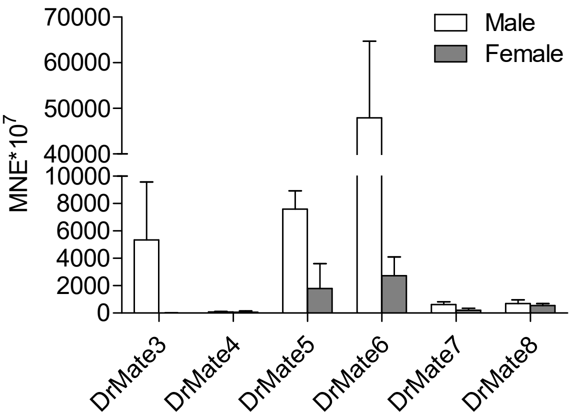

D. gills

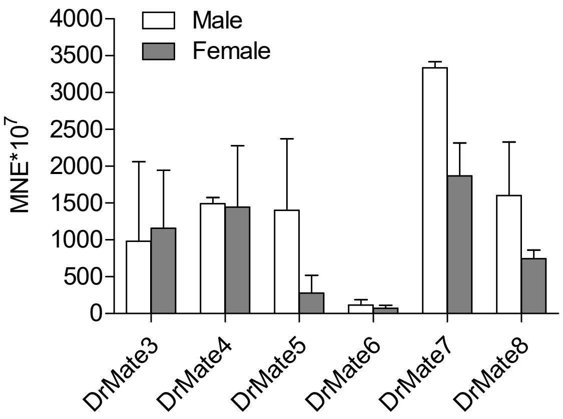

E. brain

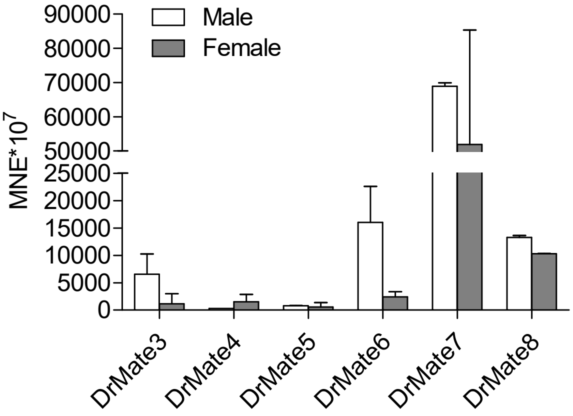

F. gonads

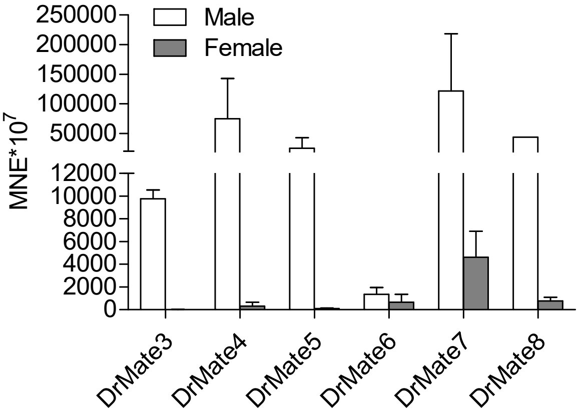

G. eye

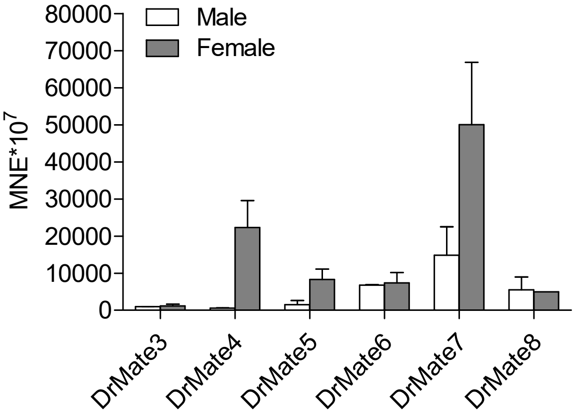

Figure S2. Tissue expression pattern of zebrafish *mate* genes: **A**, liver; **B**, kidney; **C**, intestine; **D**, gills; **E**, brain; **F**, gonads; and **G**, eye in female and male zebrafish, quantified with qRT-PCR. Results of two to three independent experiments for females and males (two to three pools of five individuals) are given, while the kidney expression data results are from two pools of 15 individuals. Data represent MNE (mean normalized expression) ± SE normalized to the Ef1α.

Figure S3.

A. Mate3

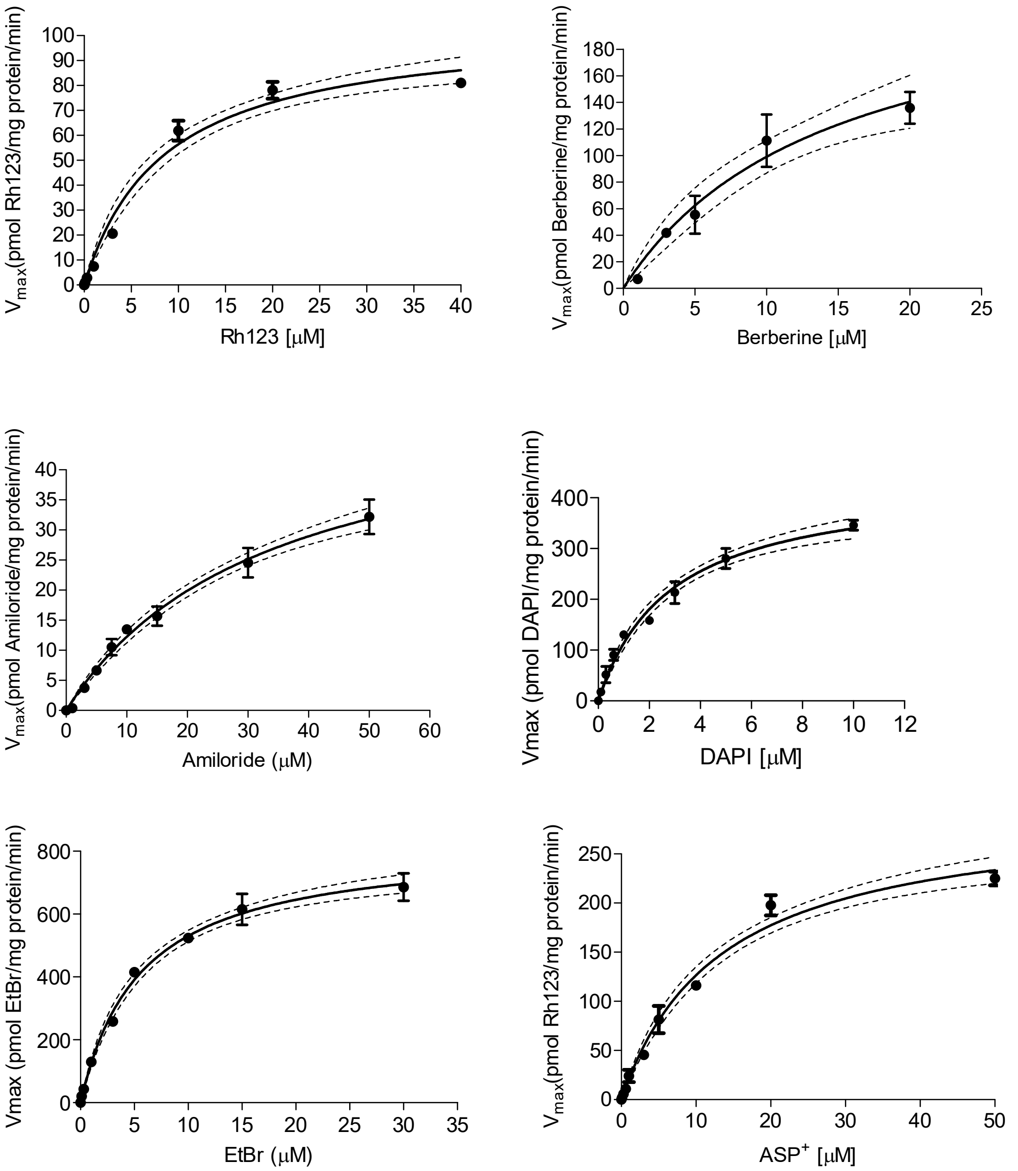

B. Mate4

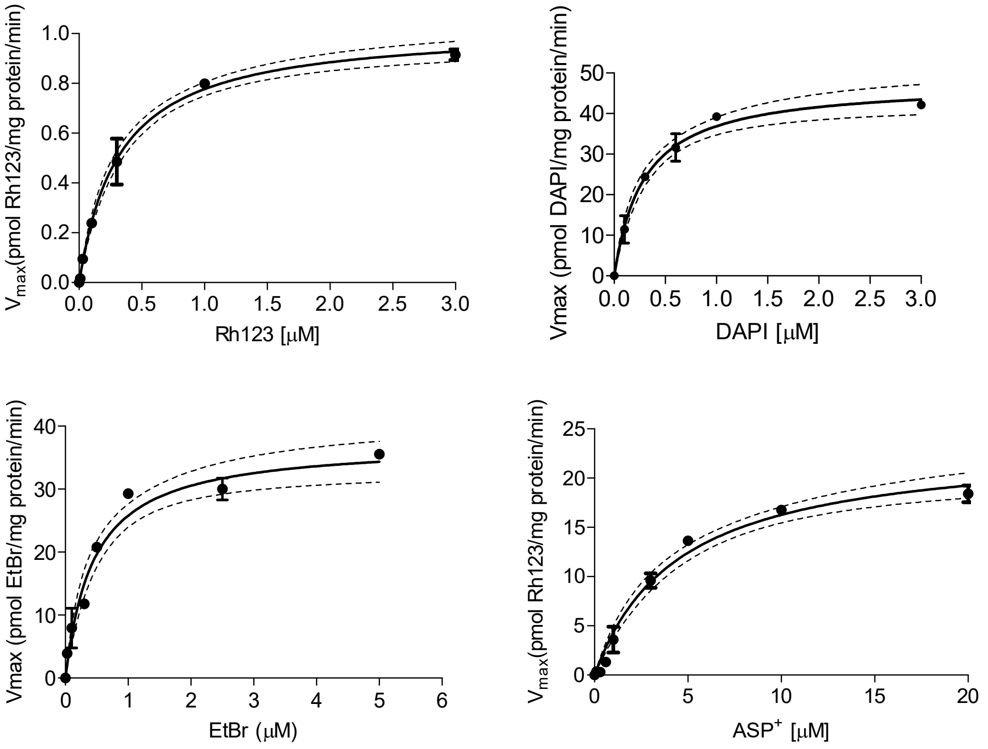

Vmax(pmol Rh123/mg protein/min)
1.0
0.8
0.6
0.4
0.2
0.0
0.0 0.5 1.0 1.5 2.0 2.5 3.0
Rh123 [μM]
Vmax (pmol DAPI/mg protein/min)
50
40
30
20
10
0
0.0 0.5 1.0 1.5 2.0 2.5 3.0
DAPI [μM]
Vmax (pmol EtBr/mg protein/min)
40
30
20
10
0
0 1 2 3 4 5 6
EtBr (μM)
Vmax(pmol Rh123/mg protein/min)
25
20
15
10
5
0
0 5 10 15 20
ASP+ [μM]

C. Mate6

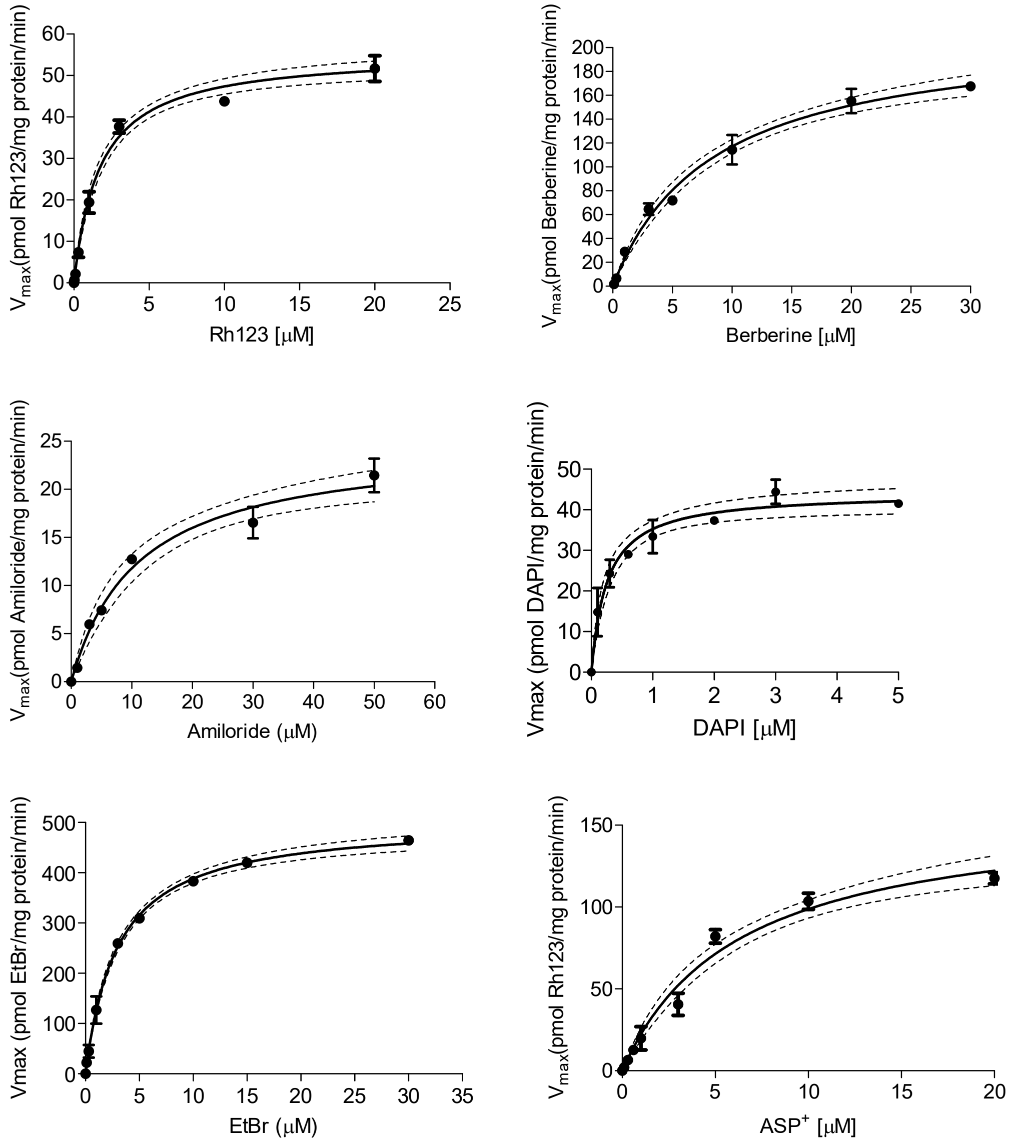

Vmax(pmol Rh123/mg protein/min)
Rh123 [μM]
Vmax(pmol Berberine/mg protein/min)
Berberine [μM]
Vmax(pmol Amiloride/mg protein/min)
Amiloride (μM)
Vmax (pmol DAPI/mg protein/min)
DAPI [μM]
Vmax (pmol EtBr/mg protein/min)
EtBr (μM)
Vmax(pmol Rh123/mg protein/min)
ASP+ [μM]

D. Mate7

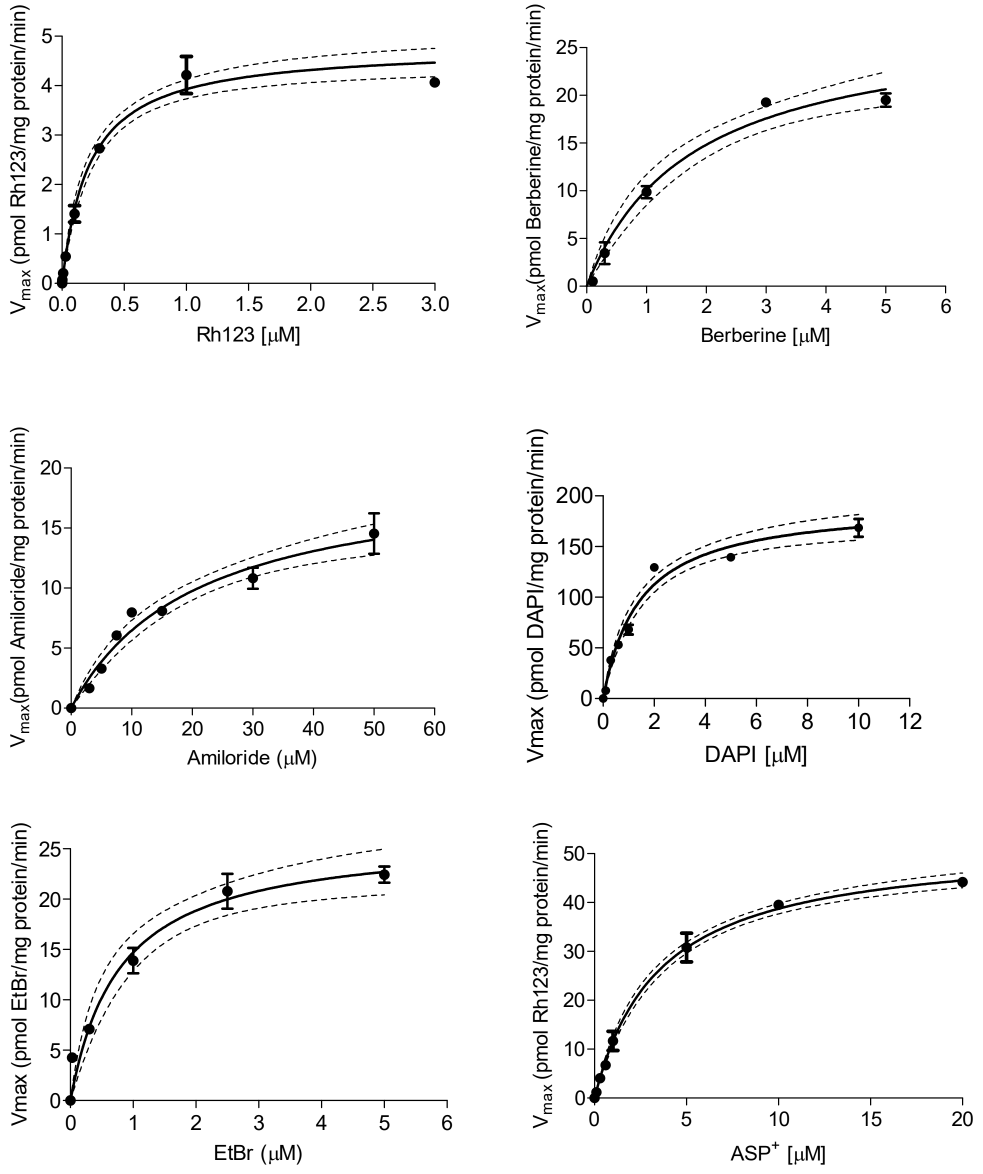
Vmax(pmol Rh123/mg protein/min)
Rh123 [μM]
Vmax(pmol Berberine/mg protein/min)
Berberine [μM]
Vmax(pmol Amiloride/mg protein/min)
Amiloride (μM)
Vmax (pmol DAPI/mg protein/min)
DAPI [μM]
Vmax (pmol EtBr/mg protein/min)
EtBr (μM)
Vmax (pmol Rh123/mg protein/min)
ASP+ [μM]

E. Mate8

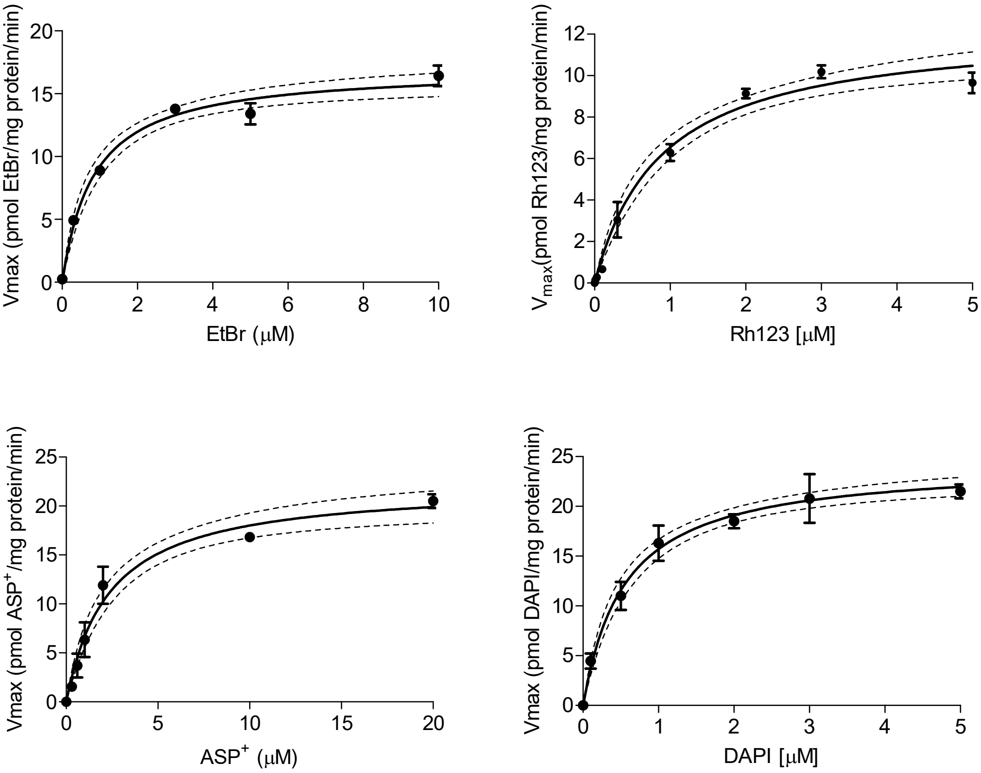

Figure S3. Michaelis-Menten kinetics of the uptake of fluorescent dyes: 4-(4-(dimethylamino)styryl)-N-methylpyridinium iodide (ASP^+), 4',6-diamidino-2-phenylindole (DAPI), rhodamine 123 (Rh123), ethidium bromide (EtBr), berberine and amiloride by zebrafish Mates: **A**, Mate3; **B**, Mate4; **C**, Mate6; **D**, Mate7 and **E**, Mate8. Concentration dependence of dye uptake expressed as transport rate (pmol/mg protein/min) over dye concentration (μM) after/during 5 min incubation with the dye except with the DAPI where kinetics for 5-10 min was measured. The uptake into vector-transfected HEK293 cells (mock cells) was subtracted to obtain transporter-specific uptake and data were fitted in the GraphPad Prism 5. Each value represents the mean + SD from triplicate determinations of a representative experiment.

Figure S4.

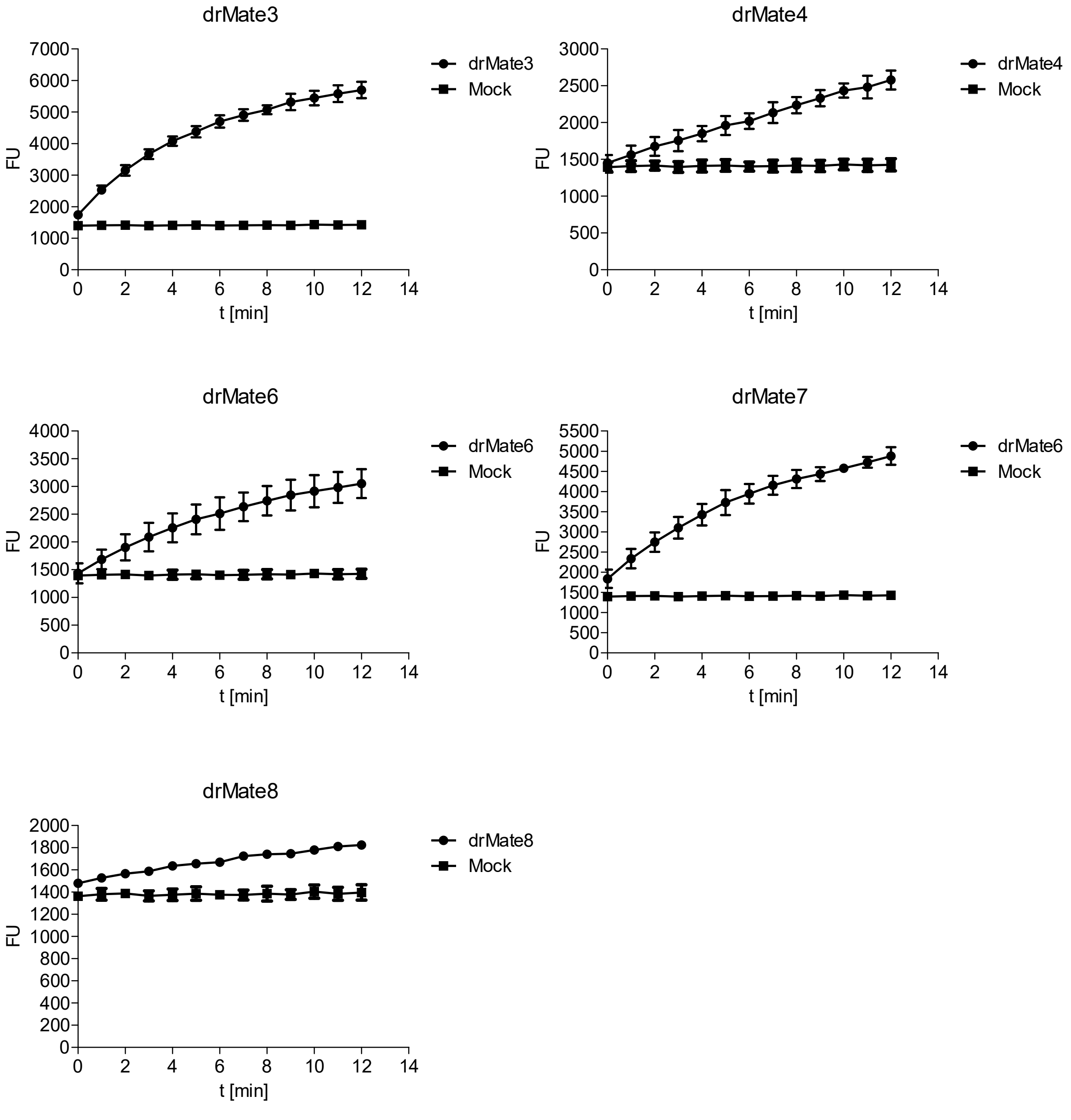

Figure S4. Time response curves for 1.0 µM DAPI (Mate4, Mate6 and Mate8) and 2.5 µM DAPI (Mate3 and Mate7).

Figure S5.

A. MPP+

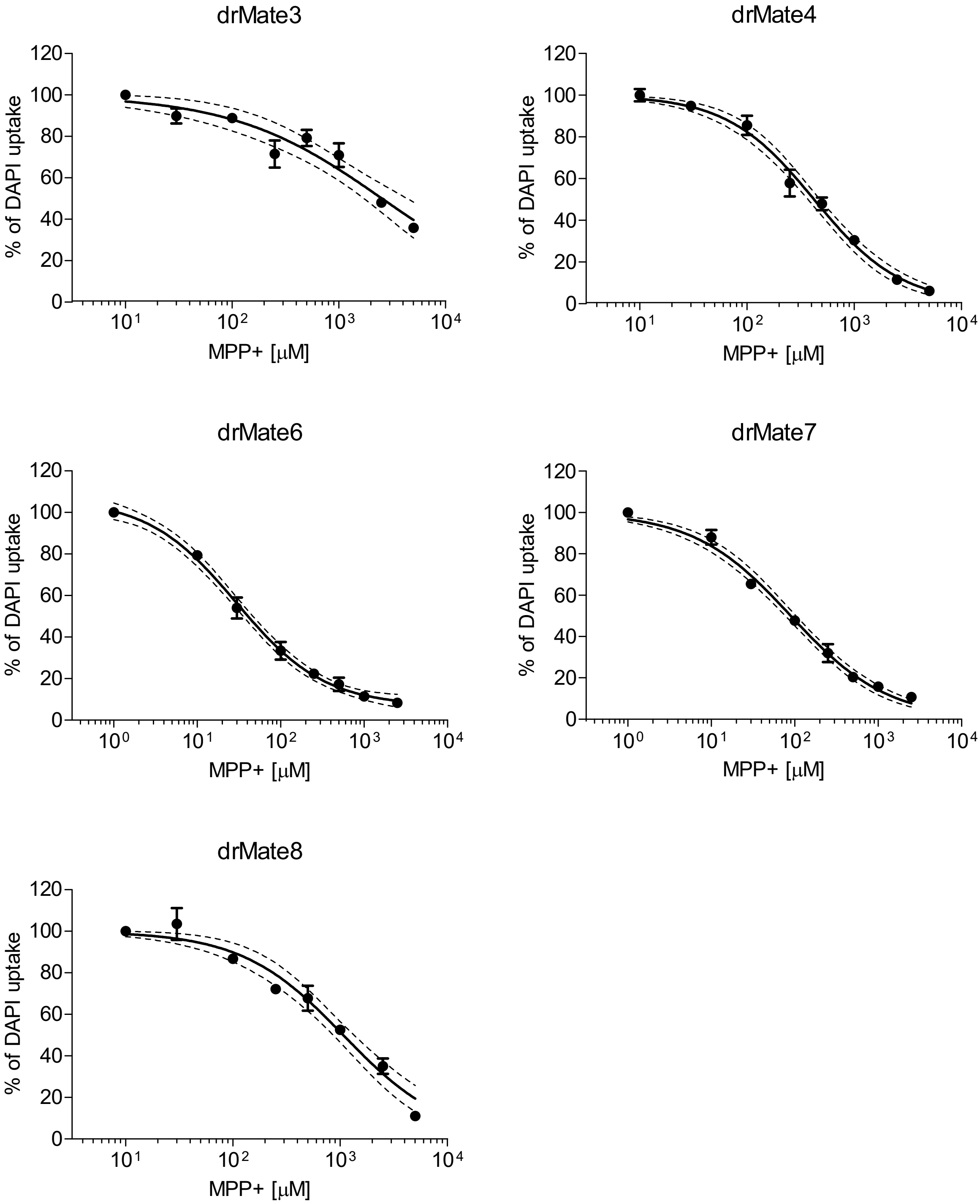

B. TEA

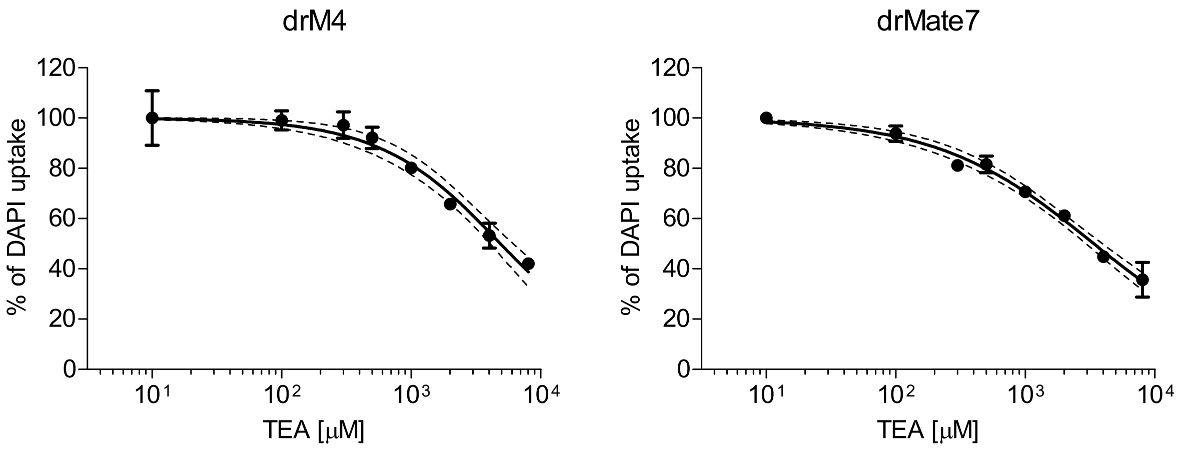
drM4
drMate7
% of DAPI uptake
TEA [μM]
0
20
40
60
80
100
120
10¹
10²
10³
10⁴

C. DHEAS

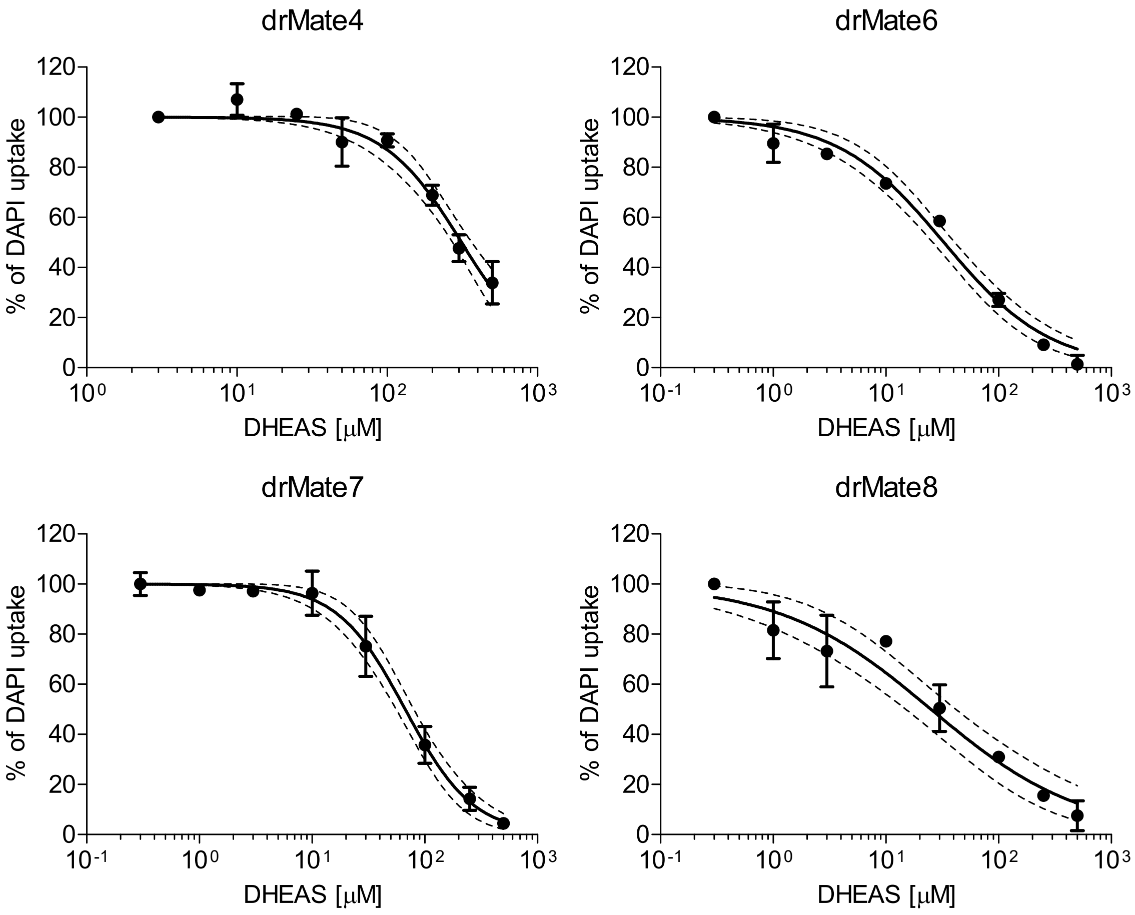
drMate4
drMate6
drMate7
drMate8
% of DAPI uptake
DHEAS [μM]
0
20
40
60
80
100
120
10⁻¹
10⁰
10¹
10²
10³

D. Testosterone

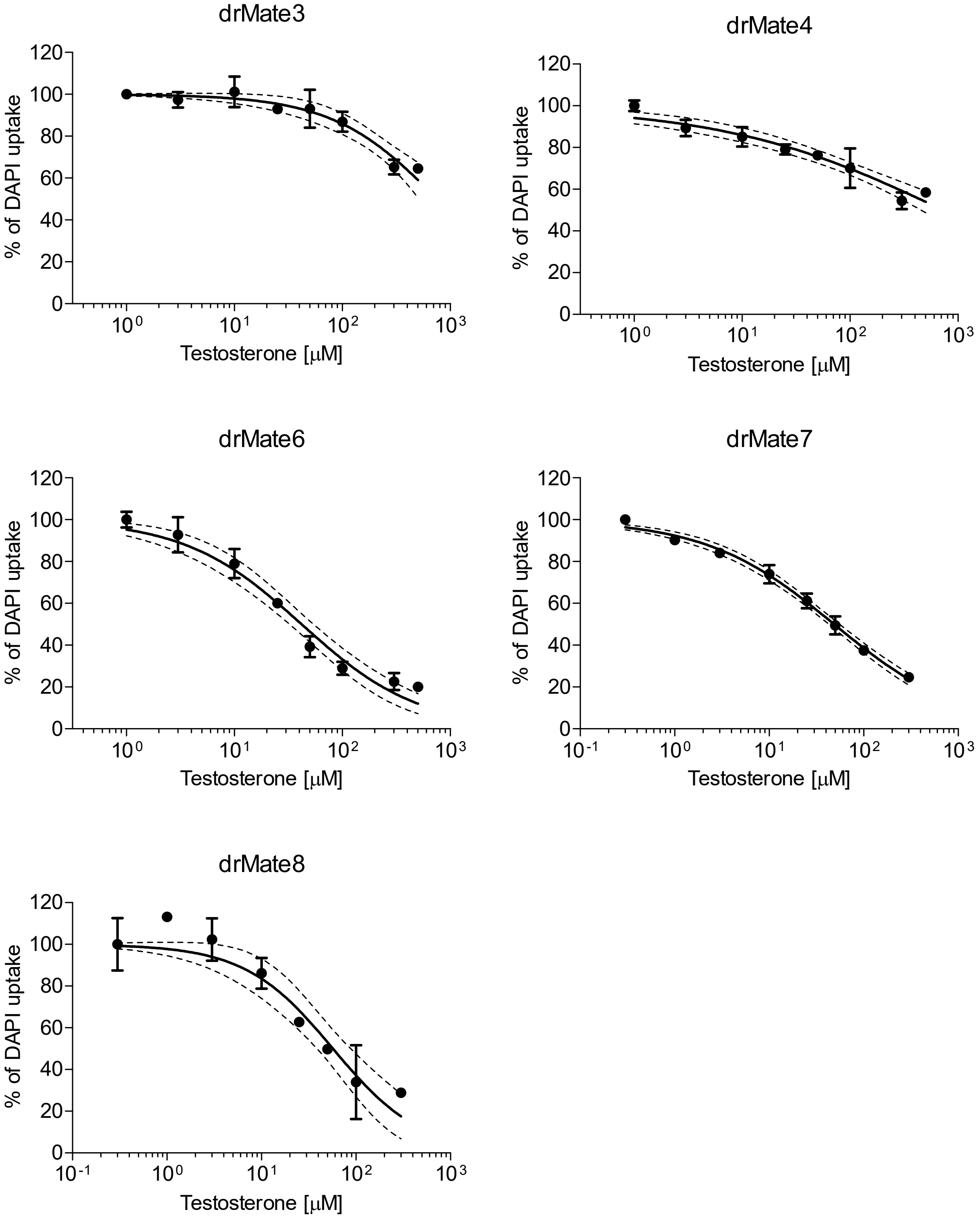

E. Androstenedione

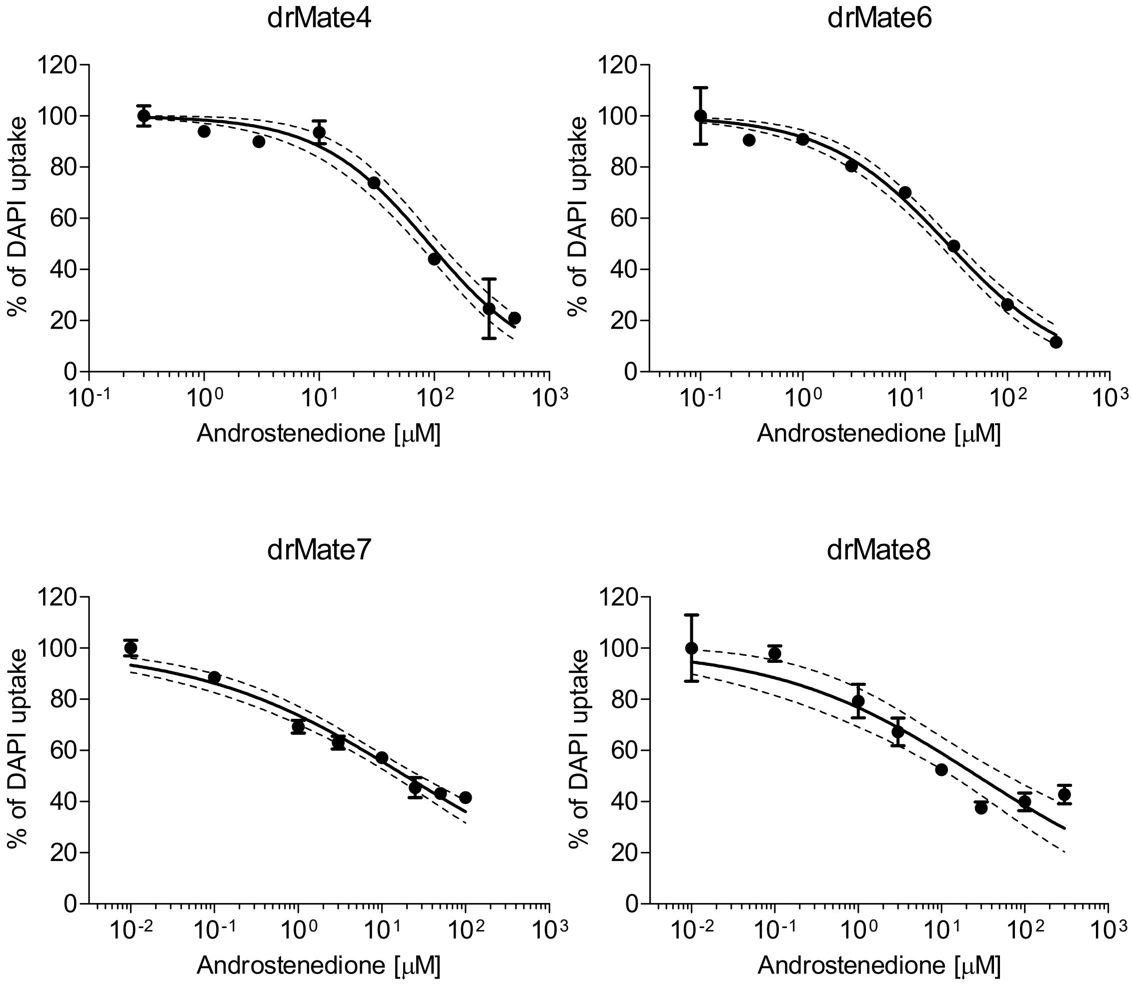

F. Deoxycholate

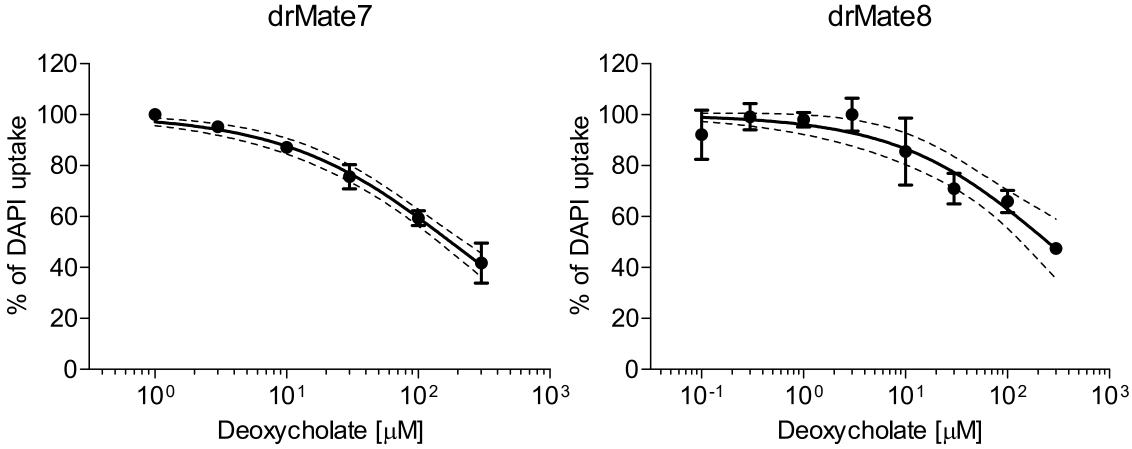

G. Corticosterone

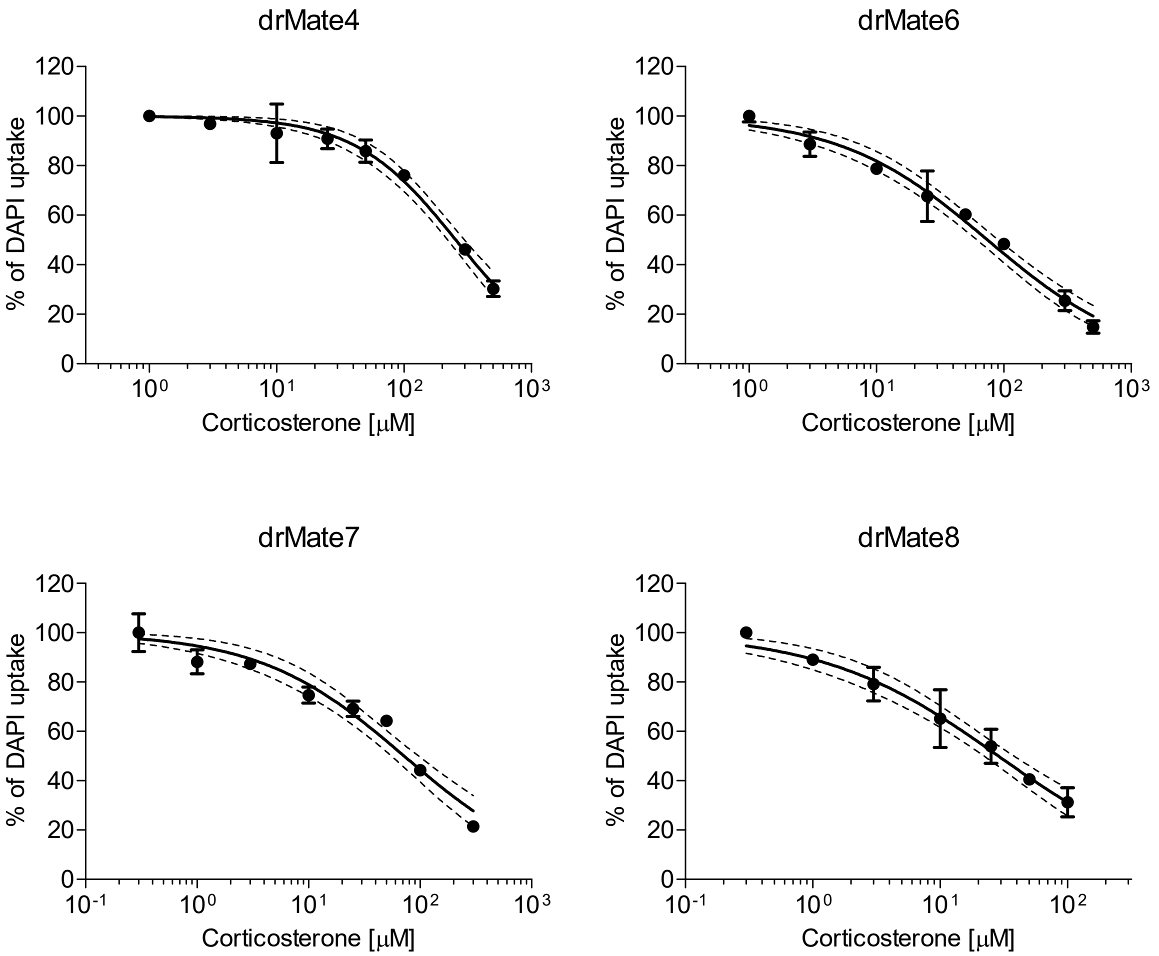

H. Thiamine

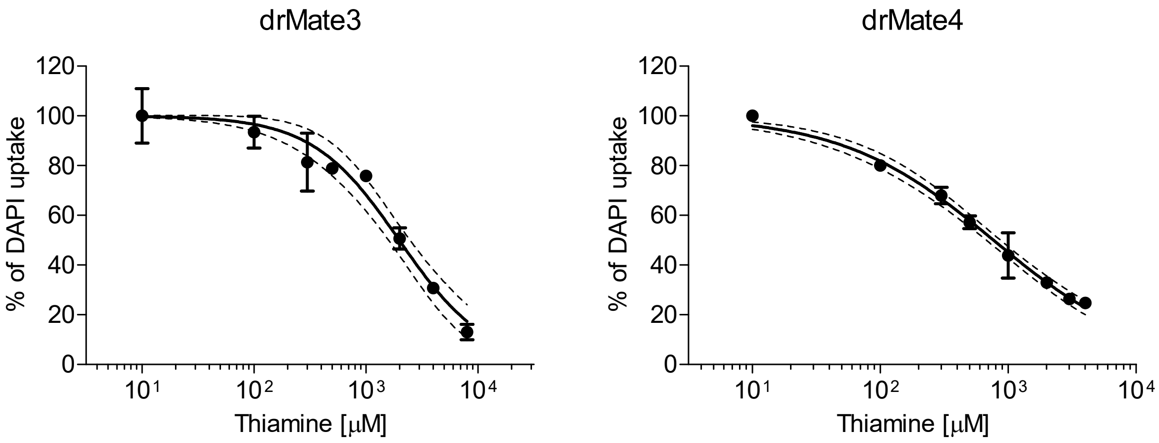

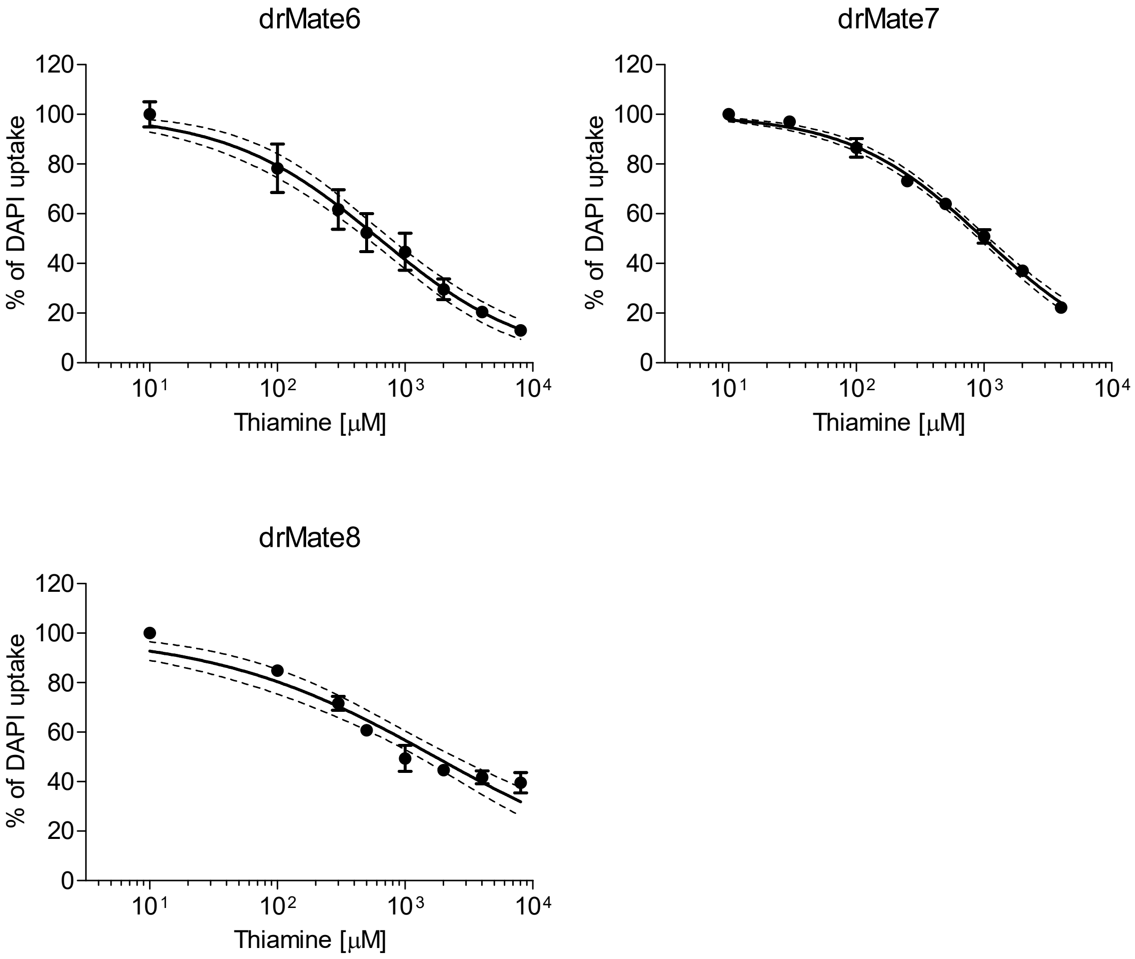

I. Cimetidine

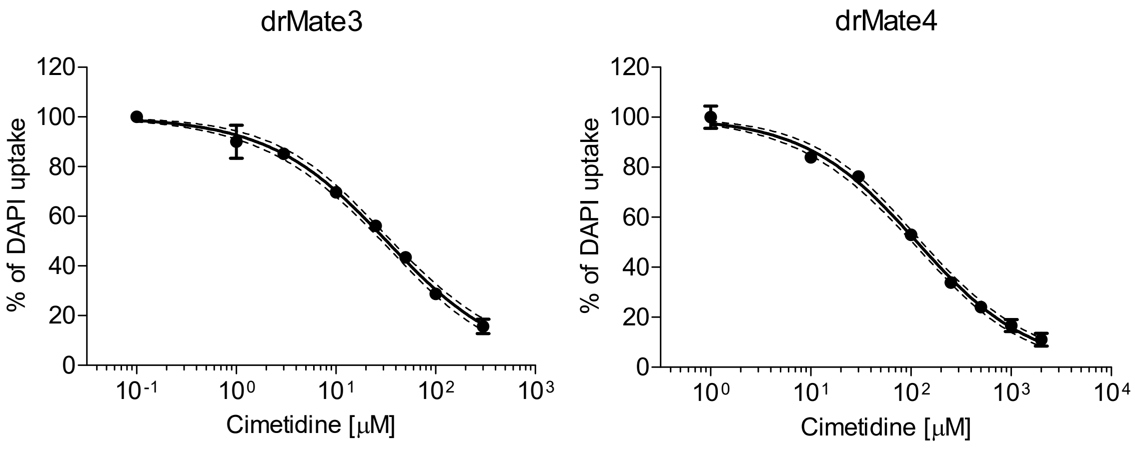

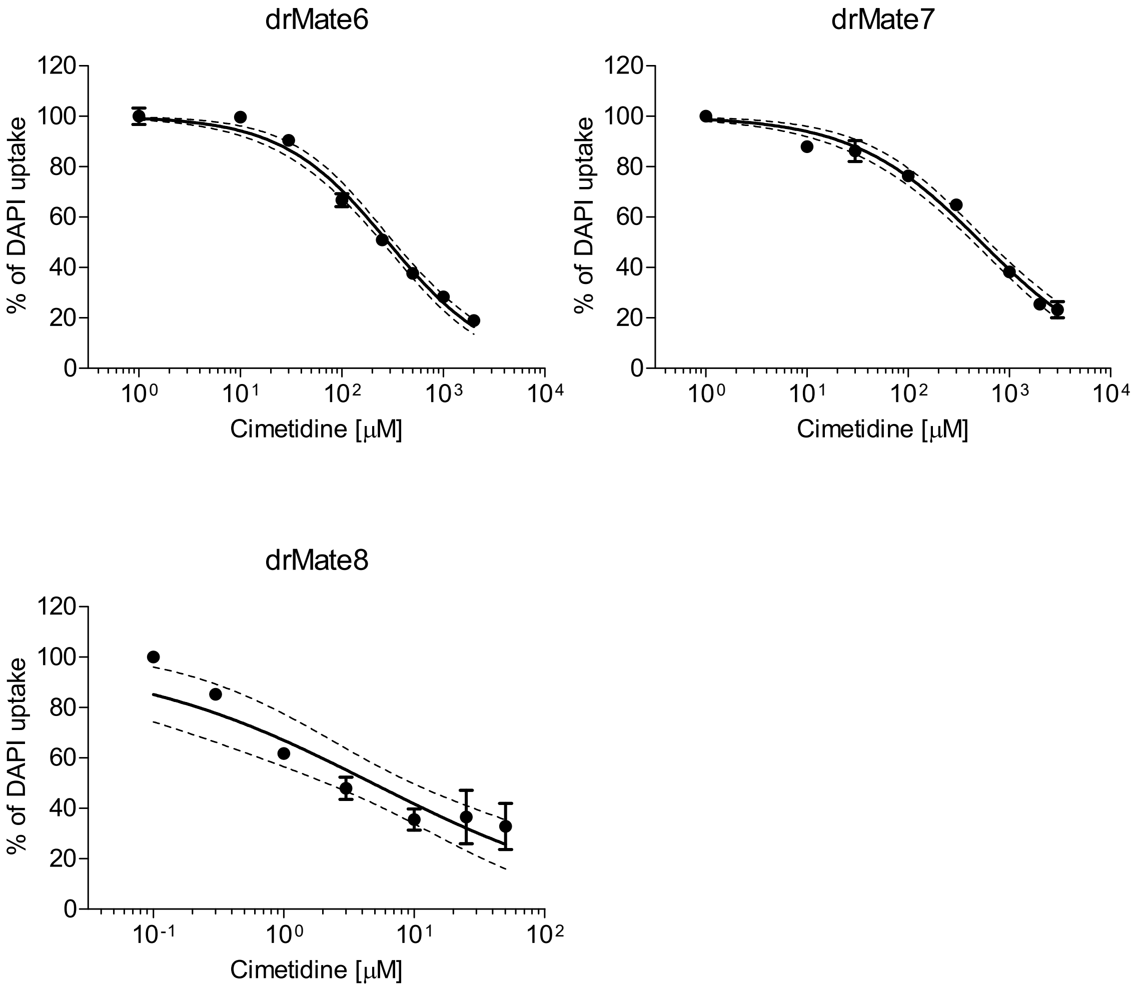
drMate6
drMate7
drMate8
% of DAPI uptake
Cimetidine [μM]

J. Verapamil

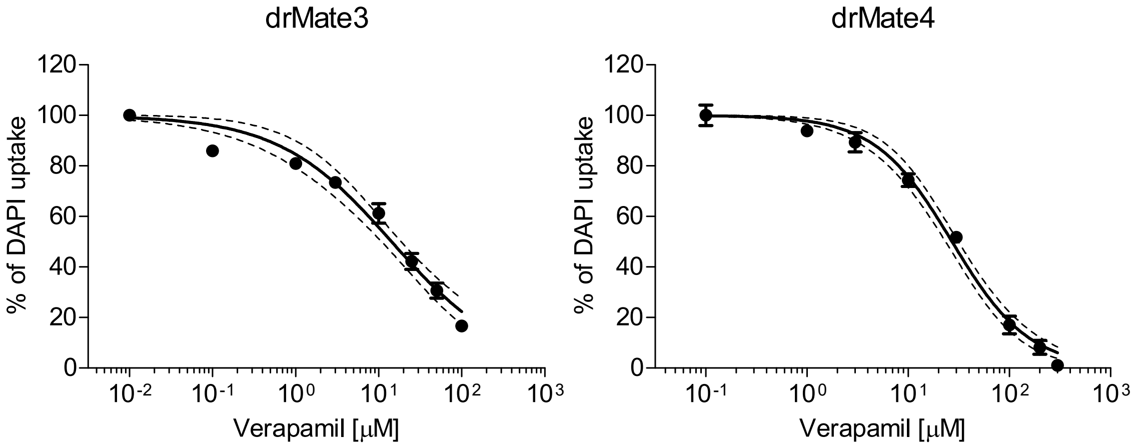
drMate3
drMate4
% of DAPI uptake
Verapamil [μM]

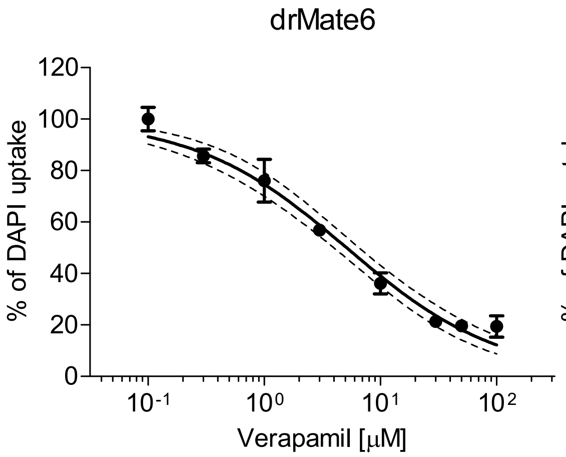
drMate6
% of DAPI uptake
Verapamil [μM]

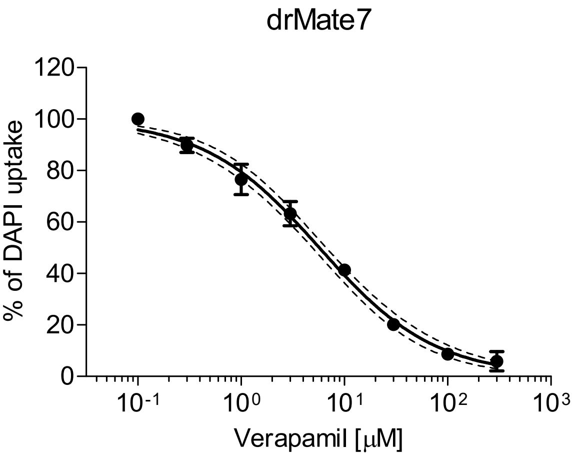
drMate7
% of DAPI uptake
Verapamil [μM]

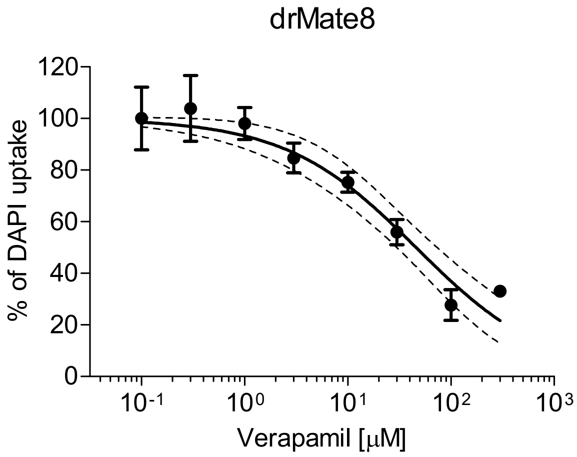
drMate8
% of DAPI uptake
Verapamil [μM]

K. Quinidine

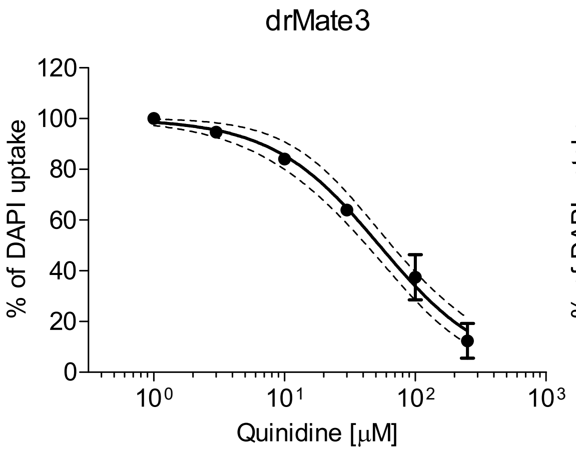
drMate3
% of DAPI uptake
Quinidine [μM]

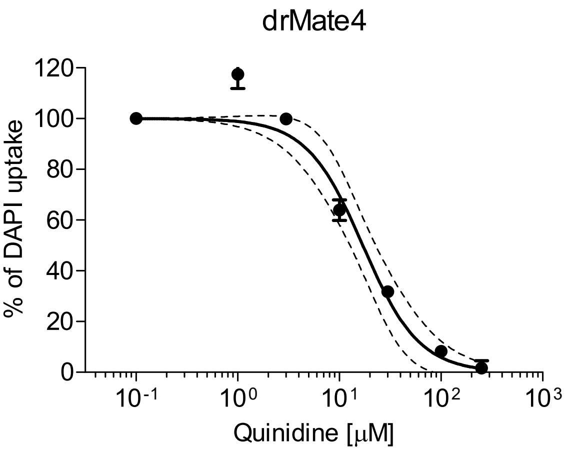
drMate4
% of DAPI uptake
Quinidine [μM]

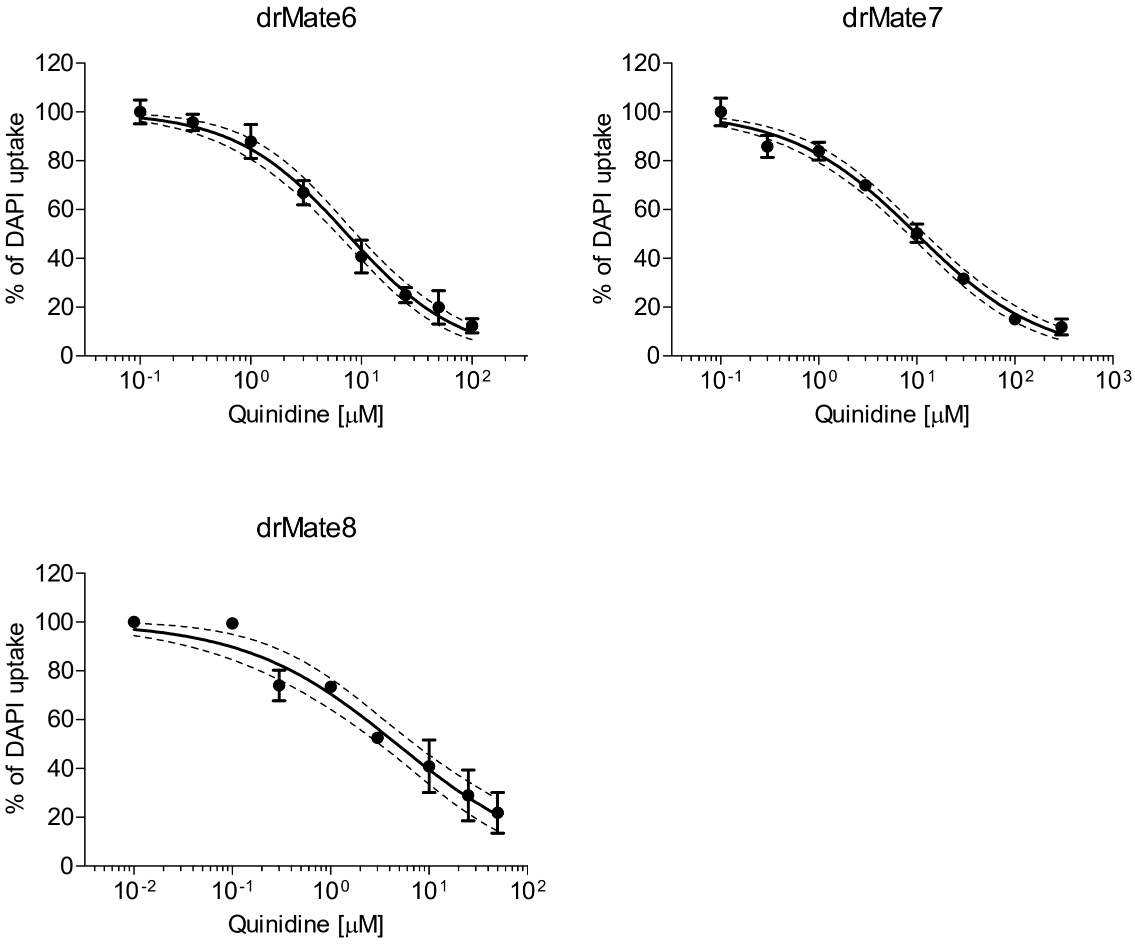
drMate6
drMate7
drMate8
% of DAPI uptake
Quinidine [μM]

L. Paraquat

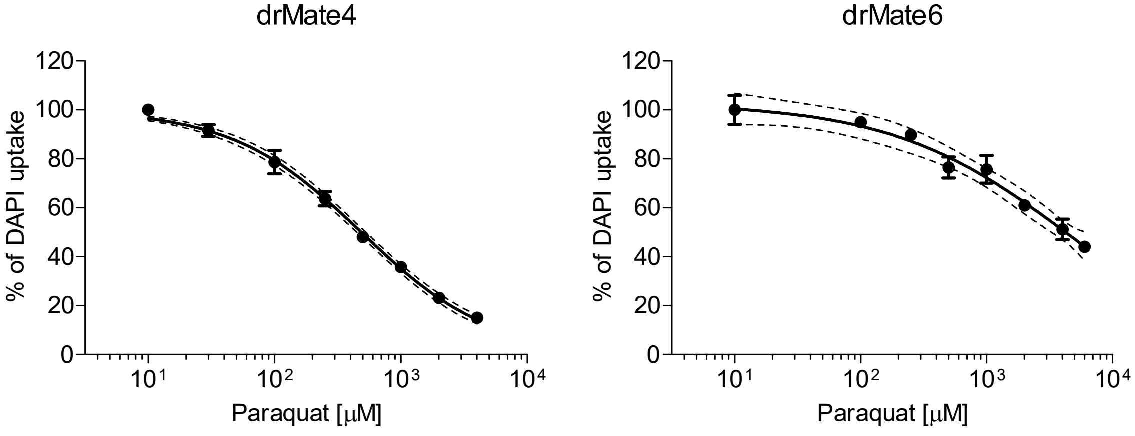
drMate4
drMate6
% of DAPI uptake
Paraquat [μM]

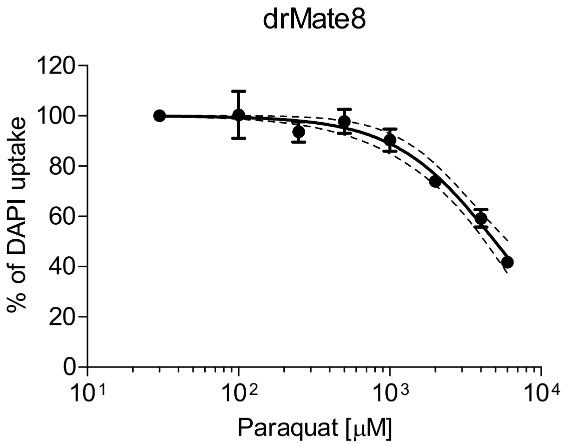

Figure S5. Concentration dependent inhibition of Mate mediated 4',6-diamidino-2-phenylindole (DAPI) uptake by typical interactors of mammalian MATEs: **A**, MPP+; **B**, TEA; **C**, DHEAS; **D**, Testosterone; **E**, Androstenedione; **F**, Deoxycholate; **G**, Corticosterone; **H**, Thiamine; **I**, Cimetidine; **J**, Veramil; **K**, Quinidine; **L**, Paraquat. The specific uptake of DAPI is expressed as a percentage relative to the DAPI uptake in the absence of an interactor (which is set to 100 %). For the IC_{50} calculations data were fitted to the sigmoidal four parameters dose–response model (variable slope) in the GraphPad Prism 5. Each data point represents the mean ± SD from triplicate determinations of a representative experiment.
